# Argonaute 2 inhibits RIG-I signaling via competition for viral RNA binding

**DOI:** 10.1016/j.isci.2025.113391

**Published:** 2025-08-19

**Authors:** Honglian Liu, Yingyin Liao, Fei Yu, Leo Ngo-Shing Li, Yajie Zhang, Lin Zhu, Guangshan Xie, Jiayan Liu, Siwen Liu, Shaofeng Deng, Rachel Chun-Yee Tam, Wenjun Song, Pin Chen, Xiaofeng Huang, Conor J. Cremin, Yixin Chen, Min Zheng, Pui Wang, Zongwei Cai, Kwok-Yung Yuen, Honglin Chen, Bobo Wing-Yee Mok

**Affiliations:** 1 Department of Microbiology, Li Ka Shing Faculty of Medicine, The University of Hong Kong, Pokfulam, Hong Kong, P.R. China; 2 State Key Laboratory for Emerging Infectious Diseases, The University of Hong Kong, Pokfulam, Hong Kong, P.R. China; 3 State Key Laboratory of Environmental and Biological Analysis, Hong Kong Baptist University, Kowloon, Hong Kong, P.R. China; 4 Centre for Virology, Vaccinology and Therapeutics Limited, The University of Hong Kong, Pokfulam, Hong Kong, P.R. China; 5 State Key Laboratory of Molecular Vaccinology and Molecular Diagnostics and National Institute of Diagnostics and Vaccine Development in Infectious Diseases, School of Life Sciences, School of Public Health, Xiamen University, Xiamen, P.R. China; 6 Department of Laboratory Medicine, The First Affiliated Hospital, Zhejiang University School of Medicine, Hangzhou, P.R. China; 7 Shenzhen Senior High School WENBO High School, Shenzhen, P.R. China; 8 Weill Cornell Medicine, New York, NY, USA; 9 Institute of Infectious Diseases, Shenzhen Bay Laboratory, Shenzhen, P.R. China

**Keywords:** Biological sciences, Microbiology, Natural sciences, Pharmacology, Virology

## Abstract

Interferon (IFN)-dependent responses constitute a critical initial defense against viruses in mammalian cells, while RNA interference (RNAi) acts as an additional strategy to combat invading viral pathogens. Investigating the functionality of mammalian Argonaute 2 (AGO2), an essential component of the RNA-induced silencing complex, we found it to negatively modulate influenza A virus infection-induced RIG-I-mediated antiviral signaling. AGO2 depletion in human cell lines significantly enhanced the RNA virus-triggered phosphorylation of IRF3 and downstream antiviral gene activation. Interestingly, this negative regulation occurred independently of gene silencing via canonical RNA silencing pathways and instead involved the binding of AGO2 to viral RNA molecules carrying 5′-triphosphates or cytosolic RIG-I agonists. These findings highlight AGO2’s crucial role in balancing antiviral signaling activation and restricting virus infection to prevent excessive immune responses.

## Highlights

• Ago2 negatively regulates RIG-I signaling beyond RNA interference
• Ago2 binds viral 5′-triphosphate hairpin RNA, suppressing immune response
• Ago2 competes with RIG-I for viral RNA binding, affecting sensing
• Ago2 balances antiviral activation and virus restriction, preventing overactivation

## Graphical abstract

**Figure:**
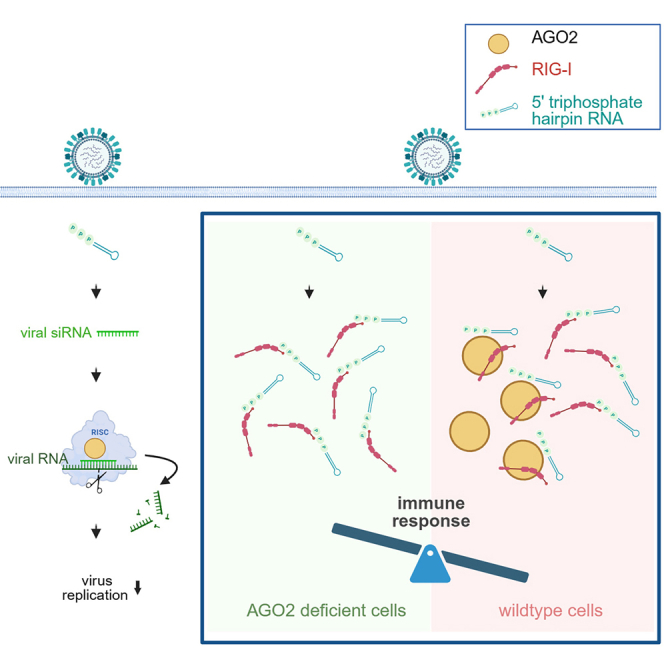

## Introduction

Human Argonaute 2 (AGO2) is a key regulator in small-RNA-guided gene silencing pathways, affected functionally by various post-translational modifications, and has a complex interplay with innate immunity.^1^ Of the four mammalian AGO proteins (AGO1-AGO4), AGO2 uniquely functions as an endonuclease for cleavage of specific sequences; however, all four can bind small RNA species, including microRNAs (miRNAs) or short interfering RNAs (siRNAs), forming the RNA-induced silencing complex (RISC).^2^^,^^3^^,^^4^ AGO2 serves as a multifunctional modulator in canonical and non-canonical miRNA- and siRNA-mediated pathways that regulate gene expression in various biological activities, as well as having other novel functions in the nucleus, including RNA activation, alternative splicing, chromatin remodeling, and double-strand break repair.^5^^,^^6^^,^^7^ AGO2 also exerts its complex function in antiviral innate immune responses through microRNA (miRNA) pathways and RNA interference (RNAi) pathways.^1^ The distinct patterns of gene expression in the interferon system and the reciprocal inhibition between miRNA and antiviral immunity may contribute to the regulation of miRNA pathways in mammalian host-virus interactions.^8^^,^^9^^,^^10^ In plants and invertebrates, RNAi serves directly as an antiviral response against virus infections, in which the RNase III endonuclease DICER catalytically cleaves viral RNA into 21–24 nt siRNAs, known as virus-derived small interfering RNAs (vsiRNAs). These vsiRNAs selectively bind to AGO2 through the complementary recognition of viral RNA, which leads to the formation of the RNA-induced silencing complex (RISC). The RISC complex then targets and cleaves the viral RNA, effectively suppressing viral replication and spreading within the host organism.^4^^,^^11^ In mammals, the ancestral RNAi antiviral system is considered to have been replaced by various pattern recognition receptors (PRRs) mediating antiviral responses.^12^^,^^13^ Recently, vsiRNAs have been detected in mammalian somatic cells during various virus infections, such as influenza A virus (IAV), Zika virus, and SARS-CoV-2^14^^,^^15^^,^^16^^,^^17^^,^^18^ but the mechanisms of biogenesis of vsiRNAs, the reality of their antiviral function in virus replication, and the relationship between RNAi and interferons (IFNs) remain inconclusive. Furthermore, the miRNA/RNAi-related effector proteins DROSHA, DICER, and AGO2 have been found to execute novel functions in host antiviral defenses that are independent of the gene silencing pathway.^19^^,^^20^

RIG-I has been recognized as one of the most important cytosolic sensors for RNA viruses.^21^ RIG-I specifically recognizes viral RNAs bearing 5′-triphosphate (5′ppp) moieties or short-type double-stranded RNAs (dsRNAs), which include genomic or subgenomic RNAs and replication intermediates from human pathogens, such as influenza A virus, Sendai virus (SeV), measles virus, and hepatitis C virus. Some chemically or enzymatically synthesized products, such as phage polymerase *in vitro* transcribed RNAs (IVT-RNA), RNAs bearing 5′-diphosphates, and short forms of poly(I:C), also act as RIG-I ligands.^22^^,^^23^^,^^24^^,^^25^^,^^26^^,^^27^^,^^28^ In the absence of viral RNA, RIG-I maintains an auto-inhibited state where its CARDs (caspase activation and recruitment domains) are sterically folded into interaction with its helicase domain and unavailable for downstream signaling activation.^29^^,^^30^^,^^31^ Upon virus infection, the auto-repressed 2CARD is released by the recognition and binding of viral RNA mediated by the C-terminal domain (CTD) and helicase domain of RIG-I, followed by the dephosphorylation of RIG-I, and then the binding of ATP.^29^^,^^32^^,^^33^ The release of 2CARD and subsequent binding of K63-linked polyubiquitin to RIG-I together induce the tetramerization of RIG-I and translocation from the RNA end to the interior.^34^ This action results in the recruitment of the mitochondrial adaptor protein MAVS in the mitochondria through homotypic CARD-CARD interaction, thus further nucleating MAVS and facilitating MAVS filament formation.^35^^,^^36^ The activation of RIG-I and MAVS functions as a central platform to recruit downstream antiviral signaling molecules, including TRAFs, TBK1, IKKε, and IRF3/7. The phosphorylation of IRF3 and IRF7 leads to the formation of their respective homodimers, translocation into the nucleus, and binding to IFN-stimulated response element motifs (ISREs) to induce the activation of the type I IFN pathway, including the expression of IFNs, cytokines, and chemokines.^37^^,^^38^^,^^39^ However, how RIG-I effectively recognizes and binds viral RNAs and how this process is modulated by host factors and viral components remains to be elucidated.

We sought to investigate how AGO2 may be involved in innate immunity; specifically, whether it can modulate IFN antiviral signaling, in addition to its RNAi properties, in response to RNA virus infection, using influenza A and Sendai viruses as models. We identified AGO2 to be a negative modulator of RIG-I-mediated antiviral signaling in responses to infection with RNA viruses. Knockout of AGO2 significantly upregulates the RNA virus-triggered phosphorylation of IRF3 and transcriptional levels of downstream antiviral genes in human epithelial cells. Mechanistically, AGO2 recognizes and associates with 5′ppp viral RNAs. We present a novel mechanism by which AGO2 regulates RIG-I signaling through competition for viral RNA association with RIG-I, whereby AGO2 interference with RIG-I viral RNA sensing attenuates RIG-I antiviral signaling. Contrarily, AGO2 binding to viral-derived siRNA, along with its role in RNAi, has been shown to restrict the replication of influenza viruses.^15^ These results highlight the complex interplay between different components of the RNA interference and innate immune systems and provide new insights into the mechanisms underlying antiviral defenses.

## Results

### Argonaute 2 negatively regulates antiviral innate immune responses

**Figure 1:**
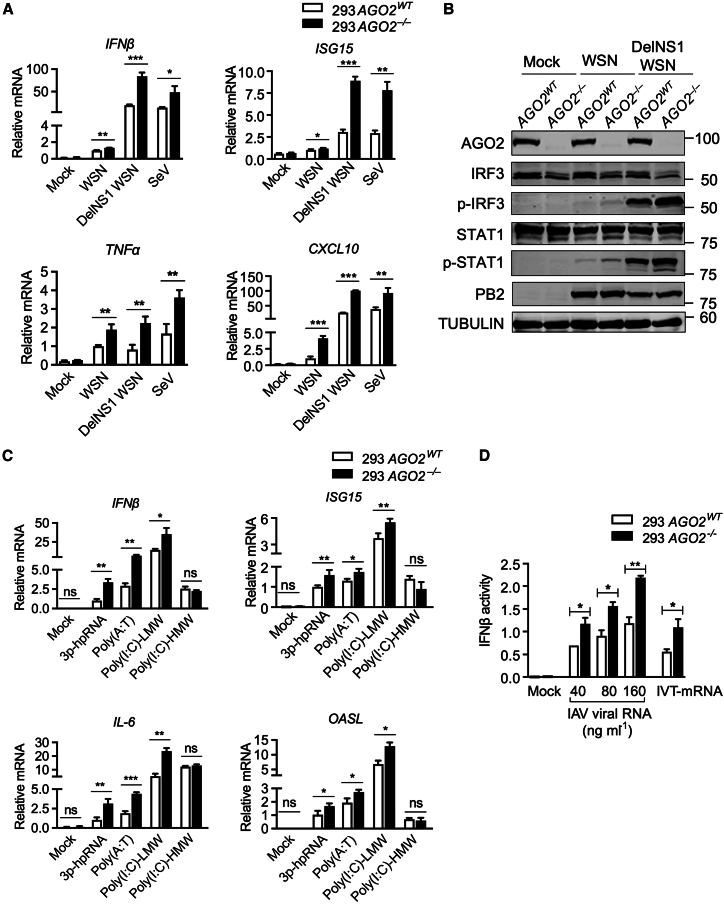
AGO2 negatively regulates RIG-I-mediated innate immune response (A) RT-qPCR analysis expression of *IFNβ*, *ISG15*, *CXCL10*, and *TNFα* mRNA in control (*AGO2*^*WT*^) and *AGO2*^−/−^ 293 cells, following either mock-infection or infection with WT WSN or DelNS1 WSN at an MOI of 1 for 8 h or SeV at an MOI of 1 for 4 h, normalized to control (*AGO2*^*WT*^) cells infected with WT WSN. (B) Immunoblot analysis of the virus-triggered phosphorylation of IRF3 and STAT1 in control (*AGO2*^*WT*^) and *AGO2*^−/−^ 293 cells following either mock infection or infection with WT WSN or DelNS1 WSN for 8 h at an MOI of 1. (C) RT-qPCR analysis of *IFNβ*, *ISG15*, *OASL*, and *IL-6* mRNA expression in control (*AGO2*^*WT*^) and *AGO2*^−/−^ 293 cells, following either mock-transfection or transfection with 3p-hpRNA, poly(A:T), poly(I:C)-LMW or poly(I:C)-HMW for 10 h, normalized to 3p-hpRNA treated control (*AGO2*^*WT*^) cells. (D) IFNβ promoter luciferase activities in control (*AGO2*^*WT*^) and *AGO2*^−/−^ 293 cells, after being mock-stimulated or stimulated with increasing concentrations of IAV WSN viral RNAs or *in vitro* transcribed (IVT) mRNA (included as a positive control). Statistical significance was analyzed using two-tailed Student’s t test. For all panels, ∗*p* < 0.05, ∗∗*p* < 0.01, ∗∗∗*p* < 0.001), ns (no significance). Error bars represent for triplicate biological experiments (mean ± SD, *n* = 3).

To explore as-yet-uncharacterized roles of AGO2 in the antiviral innate immune response during RNA virus infection, we generated *AGO2*^−/−^ 293 and A549 cell clones using CRISPR-Cas9 technology. First, control (*AGO2*^*WT*^) and *AGO2* knockout (*AGO2*^−/−^) 293 and A549 cells were infected with influenza A virus WSN (A/WSN/1933), DelNS1 WSN (deletion mutant of WSN), or Sendai virus (SeV) to induce immune responses. To circumvent any undesired interference from NS1, we opted to utilize DelNS1 WSN and SeV for the infection. This is because NS1 can interact with numerous host proteins, suppressing cellular mRNAs through RIG-I receptor interactions and competitive dsRNA binding. Additionally, NS1 can engage with various ISGs to counteract the host’s natural immune response. Quantitative RT-PCR (RT-qPCR) experiments showed that knockout of AGO2 significantly enhances the expression of mRNA for interferon β and downstream genes, including *ISG15*, *TNFα*, and *CXCL10* (Figure 1A; Figure S1A). The effect of AGO2 on *IFNβ* and related gene expression induced by different viruses was also confirmed with the knockdown of AGO2 in 293 cells (Figure S1B). To further understand the role of AGO2 in RNA virus-induced antiviral signaling, the phosphorylation status of IRF3 (*p*-IRF3) and STAT1 (p-STAT1) was estimated in *AGO2* knockout (*AGO2*^−/−^) 293 and A549 cells and in control (*AGO2*^*WT*^) cells following infection with different RNA viruses, including WSN, DelNS1 WSN and SeV (Figure 1B; Figures S1C and S1D). Immunoblot analysis demonstrated that AGO2 deficiency significantly increased the phosphorylation of IRF3 and STAT1 upon virus infection (Figure S2A). Since RIG-I is important in promoting the expression of interferons for the antiviral response, we subsequently investigated the potential involvement of AGO2 in regulating the signaling pathway of RIG-I-like receptors (RLRs). Consistent with the above results, we found that knockout of AGO2 markedly enhanced the transcription of antiviral genes, including *IFNβ*, *ISG15*, *OASL*, and *IL-6*, following treatment with different RIG-I ligands, including 3p-hpRNA (a 5′-triphosphate hairpin RNA), poly(A:T) (synthetic double-stranded DNA sequence), and poly(I:C)-LMW (low-molecular-weight poly(I:C)), but not MDA5 ligands (poly(I:C)-HMW, high-molecular-weight poly(I:C)) (Figure 1C). In another assay, IFNβ reporter activity was estimated upon the transfection of cells with purified vRNAs from influenza A virus particles. As a positive control, *in vitro* transcribed mRNA (IVT-mRNA) was used, which was generated by *in vitro* transcription from a template of the influenza M gene segment. This template included an added poly(A) tail and retained the 5′-triphosphate structure, ensuring its ability to induce IFNβ reporter activity. As expected, the IFNβ reporter was activated significantly more strongly in *AGO2*^−/−^ cells than in control cells, and in a dose-dependent manner (Figure 1D). Collectively, these results suggest that AGO2 is an important regulator of RIG-I-mediated innate immune responses induced by RNA viruses and RIG-I ligands.

### Argonaute 2 inhibition of antiviral immune responses is independent of gene silencing pathways

**Figure 2:**
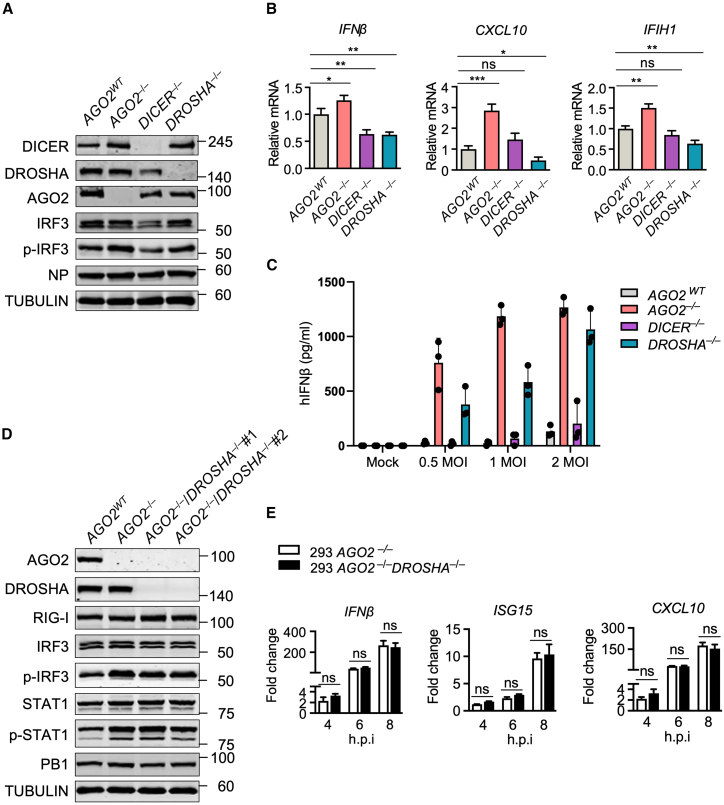
AGO2 suppresses the antiviral immune response independently of the canonical gene silencing pathway (A) Immunoblot analysis of the virus-triggered phosphorylation of IRF3 in control (*AGO2*^*WT*^), *AGO2*^−/−^, *DICER*^−/−^, and *DROSHA*^−/−^ A549 cells following infection with DelNS1 WSN for 6 h at an MOI of 1. (B) RT-qPCR analysis of *IFNβ*, *CXCL10*, and *IFIH1* mRNA expression in control (*AGO2*^*WT*^), *AGO2*^−/−^, *DICER*^−/−^, and *DROSHA*^−/−^ A549 cells after 6 h of DelNS1 WSN infection at an MOI of 1, normalized to A549 control (*AGO2*^*WT*^) DelNS1 WSN infected cells. (C) IFNβ levels in the cell supernatants after 24 h of DelNS1 WSN infection at different MOIs were measured using a Human IFNβ DuoSet ELISA Kit (R&D Systems). (D) Immunoblot analysis of the virus-triggered phosphorylation of IRF3 and STAT1 in control (*AGO2*^*WT*^), *AGO2*^−/−^, *AGO2*^−/−^*DROSHA*^−/−^#1 and *AGO2*^−/−^*DROSHA*^−/−^#2 293 cells, following 6 h of infection with DelNS1 WSN at an MOI of 1. (E) RT-qPCR time-course analysis of *IFNβ*, *CXCL10*, and *ISG15* mRNA expression in *AGO2*^−/−^ and *AGO2*^−/−^*DROSHA*^−/−^ 293 cells, after being infected with DelNS1 WSN at an MOI of 1, sampled at the indicated time points and normalized to control (*AGO2*^*WT*^) cells infected for 4 h. h.p.i., hours post-infection. Statistical significance was analyzed using two-tailed Student’s t test. For all panels, ∗*p* < 0.05, ∗∗*p* < 0.01, ∗∗∗*p* < 0.001, ns indicates no significance). Error bars represent for triplicate biological experiments (mean ± SD, *n* = 3).

**Figure 3:**
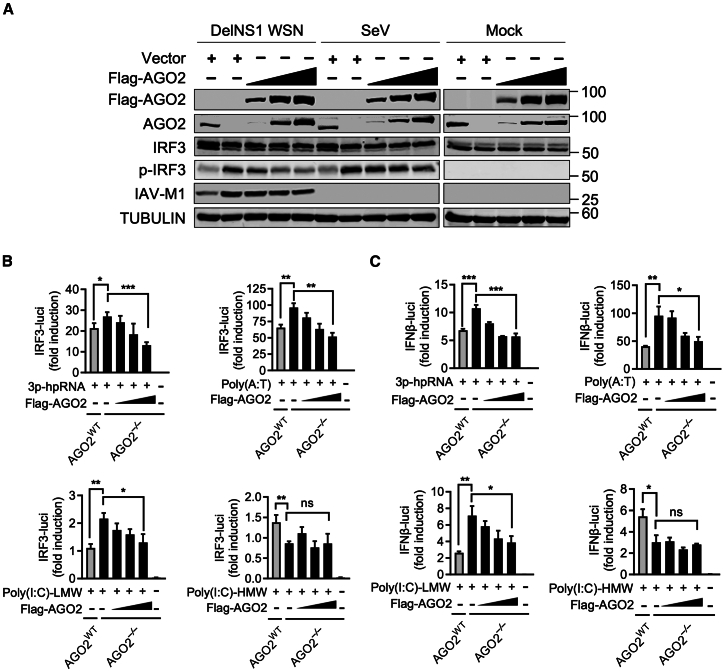
Ectopic overexpression of AGO2 impedes the RIG-I-mediated signaling pathway (A) Immunoblot analysis of the virus-triggered phosphorylation of IRF3 and STAT1 in control (*AGO2*^*WT*^) or *AGO2*^−/−^ 293 cells, after transfection with vector control or increasing amounts of Flag-AGO2 expression plasmid for 24 h, followed by either mock-infection or infection with DelNS1 WSN at an MOI of 1 for 8 h or SeV at an MOI of 1 for 4 h. The first lane for each infection condition represents control (*AGO2*^*WT*^) 293 cells, and the remaining lanes are *AGO2*^−/−^ 293 cells. (B) IRF3 promoter luciferase activities in control (*AGO2*^*WT*^) and *AGO2*^−/−^ 293 cells overexpressing Flag-AGO2, after being stimulated with different ligands (3p-hpRNA, poly(A:T), poly(I:C)-LMW or poly(I:C)-HMW). (C) IFNβ promoter luciferase activities in control (*AGO2*^*WT*^) and *AGO2*^−/−^ 293 cells overexpressing Flag-AGO2, after stimulation with different ligands (3p-hpRNA, poly(A:T), poly(I:C)-LMW and poly(I:C)-HMW). Statistical significance was assessed using a two-tailed Student’s t-test. In all panels, ∗*p* < 0.05, ∗∗*p* < 0.01, ∗∗∗*p* < 0.005, and ns indicates not significant. Error bars represent the mean ± standard deviation from three biological replicates (*n* = 3).

Two key components of the siRNA and miRNA pathways, DROSHA and DICER, have been shown to have different functions in the innate immune response. A recent study shows that the deletion of DROSHA has no effect on the response of the IFN-I system.^19^ To verify that the adverse impact of AGO2 on the immune response triggered by RNA viruses is not reliant on the miRNA or siRNA pathways, our first course of action was to utilize CRISPR-Cas9 technology to generate *DROSHA*^−/−^ and *DICER*^−/−^ A549 cells. Immunoblot analysis and RT-qPCR analyses showed a higher IRF3 phosphorylation level or an increase in the transcription of interferon-associated genes in virus-infected cells in the absence of AGO2, while there were no similar effects in DROSHA or DICER knockout cells (Figures 2A and 2B; Figure S2B). The increased RNA levels in *AGO2* knockout cells correlated with enhanced IFNβ secretion, as demonstrated by ELISA at different viral doses (Figure 2C). Notably, although the depletion of DROSHA did not affect *IFNβ* mRNA levels, it influenced protein production, which may be attributed to altered miRNA profiles. This, in turn, could affect the regulation of IFN production and other antiviral responses. We then generated *AGO2*^−/−^ *DROSHA*^−/−^ double knockout 293 cells using the same procedure. As expected, increased levels of phosphorylation of IRF3 and STAT1 were found in *AGO2* knockout cells after infection with DelNS1 WSN virus, regardless of the presence of DROSHA (Figure 2D; Figure S2C). Similarly, no significant change in the expression of related antiviral genes was observed between *AGO2*^−/−^ and *AGO2*^−/−^ *DROSHA*^−/−^ cells (Figure 2E). These results are in strong agreement with another report that the loss of DROSHA has no direct effect on virus-triggered immune responses.^19^ Restoring AGO2 expression in *AGO2*^−/−^ 293 cells through the ectopic overexpression of Flag-AGO2 showed that the phosphorylation of IRF3 in response to DelNS1 WSN and SeV infections decreased in a dosage-dependent manner (Figure 3A; Figure S3A). These results revealed a potential function of AGO2 as a negative regulator of interferon signaling that acts independently of the classical miRNA or siRNA pathways. Similarly, the ectopic expression of Flag-AGO2 only affected the activation of IFNβ and IRF3 promoter-driven luciferase by RIG-I ligands (3p-hpRNA, poly(A:T), and poly(I:C)-LMW) in a dose-dependent manner, but not that triggered by MDA5 ligands (poly(I:C)-HMW), suggesting that AGO2 specifically inhibits the RIG-I/IFN signaling pathway (Figures 3B and 3C; Figure S3B).

### Interaction with viral RNA is essential for interference of the RIG-I-mediated signaling pathway by Argonaute 2

**Figure 4:**
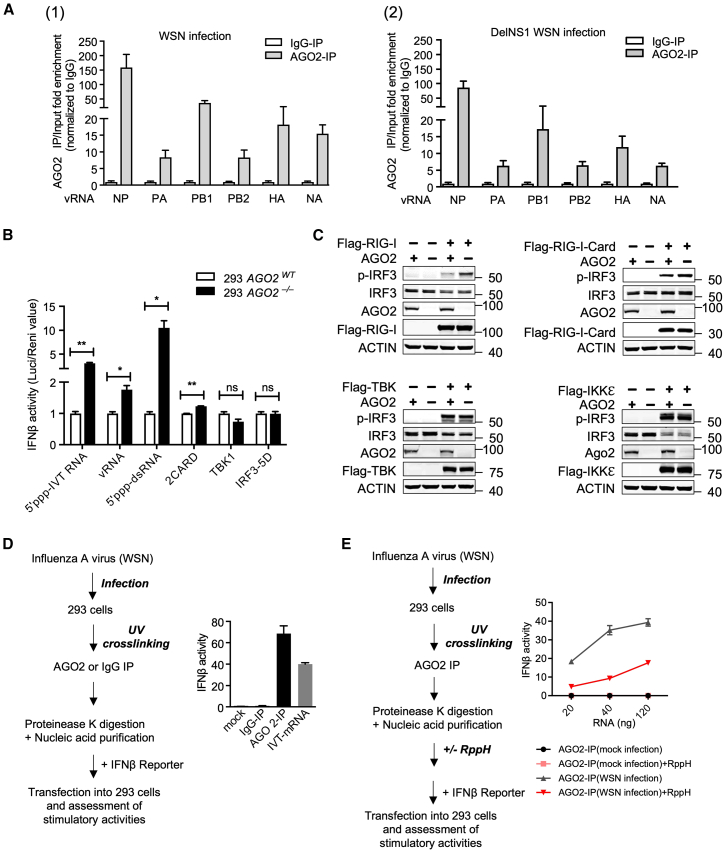
AGO2 associates with viral RNA bearing 5′-triphosphates (A) CLIP analysis of viral genome RNA conducted by endogenous AGO2 in 293 cells infected with WSN (1) or DelNS1 WSN (2) at an MOI of 1 for 8 h (*n* = 3). (B) IFNβ promoter luciferase activities in control (*AGO2*^*WT*^) and *AGO2*^−/−^ 293 cells, after stimulation with RLR ligands (5′ppp-IVT RNA, IAV viral RNA or 5′ppp-dsRNA) and some downstream effectors (RIG-I-2CARD, TBK or IRF3-5D). (C) Impact of AGO2 depletion on virus-triggered IRF-3 phosphorylation in the presence of different key signaling components involved in the production of type-I interferons. Control (*AGO2*^*WT*^) and *AGO2*^−/−^ 293 cells were overexpressed with different key signaling components involved in the production of type-I interferons. The effect on IRF3 phosphorylation was assessed using Western Blot. (D) Left, experimental procedure; right, IFNβ promoter luciferase activities with AGO2 precipitates. As a positive control, *in vitro* transcribed mRNA (IVT-mRNA) was employed (see Figure 1D for details). (E) Left, experimental procedure; right, IFNβ promoter luciferase activities with the increased concentration of AGO2 precipitates obtained from mock or WSN infected 293 cells, treated ± RppH. Statistical significance was analyzed using a two-tailed Student’s t test. For all panels, ∗*p* < 0.05, ∗∗*p* < 0.01 and ns indicates no significance. Data presented in panel B were obtained from biological duplicates (mean ± SD (*n* = 2)). These experiments were independently performed three times, with each trial showing a similar trend across all replicates.

As one of the important components of the RNA-induced silencing complex (RISC), AGO2 recognizes and binds to small RNAs and targeted mRNAs. To further understand the association of AGO2 with influenza virus mRNA and vRNA, AGO2 CLIP (crosslinking and immunoprecipitation) assays were performed in both 293 and A549 cells. UV cross-linking was used to preserve interactions between RNAs and proteins, as previously described, followed by qPCR.^40^ To differentiate between vRNA and viral mRNA, we utilized uni12 and polyA primers, respectively, for cDNA synthesis during the reverse transcription process. Our findings demonstrated that both viral vRNA and viral mRNA could be effectively enriched in AGO2 immunoprecipitates within 293 or A549 cells infected by either WSN strain, as illustrated in Figure 4A; Figure S4A). Furthermore, we conducted a similar experiment in DICER KO 293T cells (a gift from Prof. Bryan Cullen),^41^ confirming that the association between AGO2 and vRNA is independent of miRNA (Figure S4B). In an attempt to identify the target through which AGO2 suppresses interferon responses in the virus infection model, we evaluated IFNβ promoter-driven luciferase activities induced by RLR signaling ligands, including 5′ppp-IVT RNA, viral RNA, 5′ppp-dsRNA, or downstream effectors in the RIG-I pathway, including 2CARD, TBK1, IRF3, and IRF3-5D, using AGO2 knockout 293 cells (Figure 4B). Compared to control cells, knockout of AGO2 only affected IFNβ activity induced by RNA effectors bearing or producing 5′-triphosphate but for the most part had no effect on that induced by the downstream effectors. Although there was a significant difference in the induction by 2CARD in AGO2 KO cells, the level of difference was marginal (less than 1-fold). Similarly, we observed no substantial effect on IRF3 phosphorylation in AGO2 KO cells induced by 2CARD, TBK1, and IKKε. This suggests that AGO2 levels do not significantly affect the induction of IFNβ activity by downstream effectors of RIG-I (Figure 4C).

To further verify if AGO2 directly associates with viral genomic RNA, nucleic acids associated with endogenous AGO2 in influenza A virus-infected cells were immunoprecipitated and transfected into 293 cells to evaluate their stimulation of IFNβ promoter-driven luciferase activities (Figure 4D, left panel). The activation of IFNβ was only observed with the transfection of nucleic acids precipitated by AGO2-specific antibody, but not with those from control IgG immunoprecipitation (Figure 4D, right). To further confirm that the RNA species in AGO2 immunoprecipitates that induce IFNβ activities are 5′ppp viral RNAs, purified AGO2-immunoprecipitate RNAs were treated with RNA 5′ Pyrophosphohydrolase (RppH) to remove 5′-phosphates. Notably, the IFNβ stimulatory activity of these RNAs was dramatically attenuated after treatment with RppH (Figure 4E), demonstrating that AGO2 directly binds to viral genomic RNAs carrying a 5′-triphosphate moiety. Taken together, these experiments suggest that AGO2 binds to viral genomic RNA and viral mRNA, and that the recognition of viral genomic RNA by AGO2 is essential for blocking RIG-I-mediated signaling for antiviral innate immune responses at an early stage.

### Argonaute 2 competes with RIG-I for binding to viral RNA bearing 5′-triphosphates and hairpin structures

**Figure 5:**
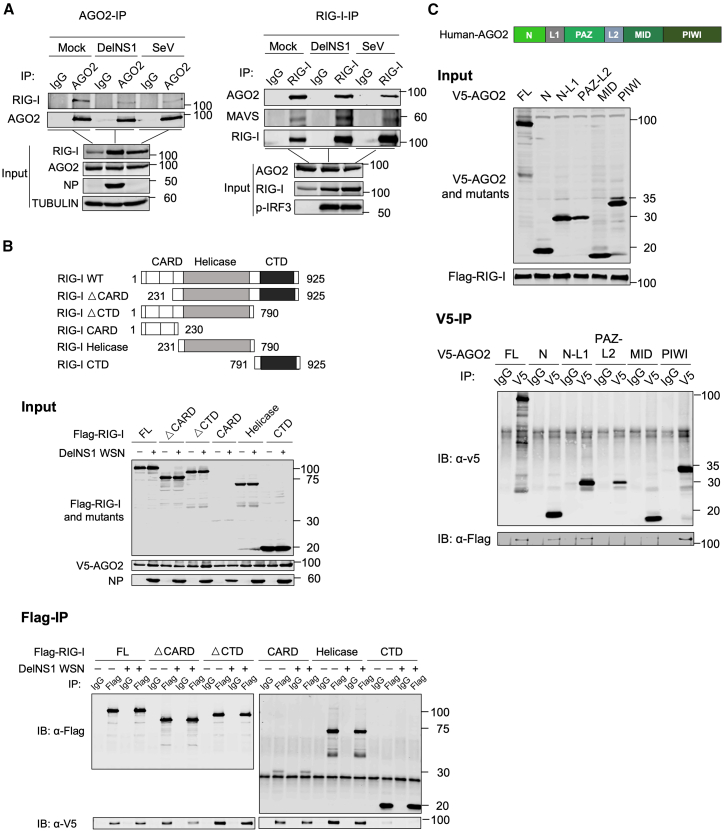
AGO2 interacts with RIG-I at endogenous and exogenous levels (A) Co-immunoprecipitation analysis of endogenous AGO2 and RIG-I in A549 cells following mock infection or infection with DelNS1 WSN at an MOI of 1 for 8 h or SEV at an MOI of 1 for 4 h: Left: AGO2 immunoprecipitation; right, RIG-I immunoprecipitation. (B) Co-immunoprecipitation analysis between V5-AGO2 and full-length (FL) and truncated versions of Flag-RIG-I in 293 cells infected with or without DelNS1 WSN at an MOI of 1 for 8 h. Upper: schematic illustration of RIG-I domain mutants; Middle: Input analysis of AGO2 and RIG-I; Lower: Co-immunoprecipitation analysis between V5-AGO2 and full-length and truncation mutants of Flag-RIG-I. (C) Co-immunoprecipitation analysis between Flag-RIG-I and full-length and truncation mutants of V5-AGO2 in 293 cells. Upper: schematic illustration of AGO2 domains; Middle: Input analysis of AGO2 and RIG-I; Lower: Co-immunoprecipitation analysis between Flag-RIG-I and full-length and truncates of V5-AGO2.

RIG-I consists of three classical domains: CARDs, helicase, and the CTD. The helicase and CTD domains are important for the recognition of viral RNA; the CTD subdomain binds directly to RNA, while the helicase domain enhances RNA binding and also plays a role in ATP hydrolysis. Several studies have characterized the RNA binding properties of RIG-I proteins, including the crystal structure of the RIG-I CTD bound to dsRNA. The RIG-I CTD binds to dsRNA in a sequence-independent manner, with the RNA binding site consisting of a positively charged groove that interacts with the phosphate backbone of the RNA. Other studies have used mutational analysis and biochemical assays to further elucidate the RNA binding properties of RIG-I proteins.^29^^,^^42^^,^^43^ We showed that AGO2 may modulate the RIG-I signaling pathway by interfering with the RIG-I detection of viral RNA. To explore the molecular basis of the AGO2 suppression of RIG-I signaling, we first examined the interaction between RIG-I and AGO2 under different infection conditions in A549 cells (Figure 5A). RIG-I was co-precipitated with AGO2 in mock-infected cells, and this association was decreased in DelNS1 WSN and SeV infections (Figure 5A, left panel). Similarly, AGO2 was detected in the reciprocal immunoprecipitation of RIG-I, and this interaction was also reduced upon SeV infection (Figure 5A, right panel). However, its interaction with other partners, such as DICER, which is not expected to be modulated by viral infection, remains unaffected under infection conditions (Figure S5). To further analyze which domains account for RIG-I interaction with AGO2, reciprocal domain mapping experiments were conducted using various truncated versions of RIG-I and AGO2. We observed that AGO2 had strong interactions with the CARD and helicase domains of RIG-I, but only weakly binds to the CTD domain, with all of these associations being slightly decreased in DelNS1 WSN virus infection (Figure 5B). Domain mapping analysis of AGO2 found that only the N and PIWI domains of AGO2 efficiently immunoprecipitated RIG-I protein (Figure 5C). The C-terminal domain (CTD) of RIG-I is responsible for recognizing both double-stranded RNA (dsRNA) and 5′ppp single-stranded RNA (ssRNA), while the MID and PAZ domains of AGO2 bind to small RNA molecules.^23^^,^^25^^,^^29^^,^^44^^,^^45^^,^^46^ Coincidentally, our findings indicate that these RNA binding domains do not directly interact with each other in RIG-I-AGO2 binding. This suggests that the physical association between AGO2 and RIG-I is independent of RNA binding, as further demonstrated by immunoprecipitation coupled with RNase treatment (Figure S6). Instead, the interaction of RIG-I and AGO2 occurs through other domains, creating the opportunity for them to compete for viral RNA binding.

**Figure 6:**
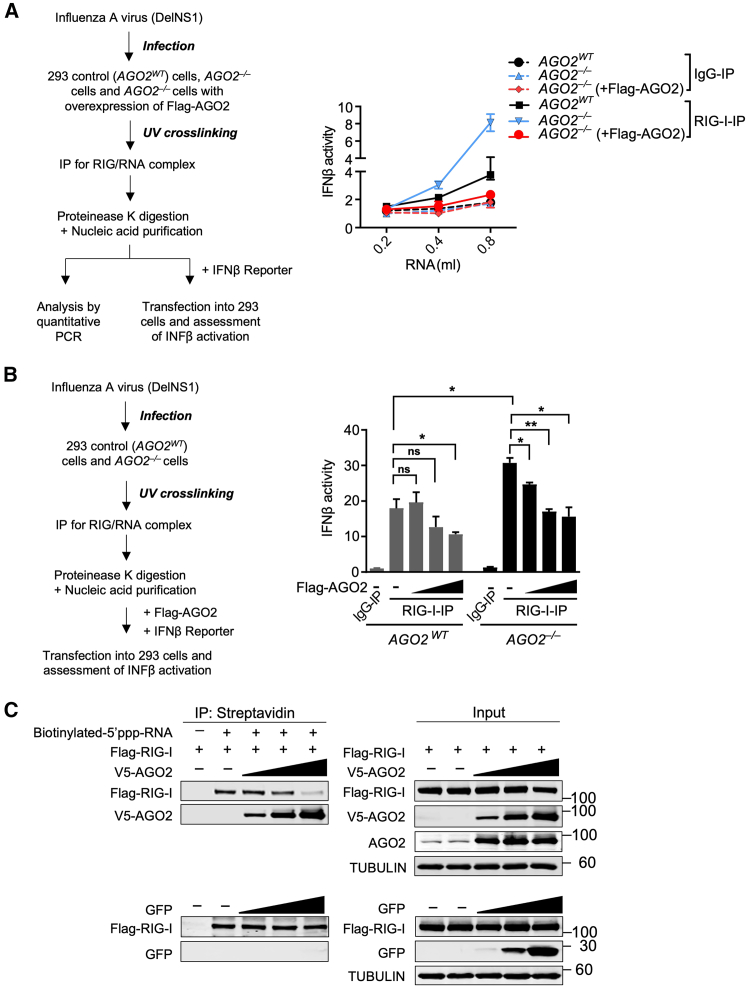
AGO2 competes with RIG-I for sensing viral RNA bearing 5′-triphosphates (A) Left, experimental procedure; right, IFNβ promoter luciferase activities with RIG-I precipitates from 293 control (*AGO2*^*WT*^) cells, *AGO2*^−/−^ cells, and *AGO2*^−/−^ cells with the overexpression of Flag-AGO2. The dashed line: control IgG immunoprecipitation; the solid line: RIG-I immunoprecipitation. Presented data are derived from biological duplicates (mean ± SD, *n* = 2), with three independent experiments showing consistent trends across all replicates. (B) Left, experimental procedure; right, RNA from control and *AGO2* knockout infected cell lines was transfected. This RNA had been immunoprecipitated using an RIG-I antibody or IgG as a control. At the same time, escalating doses of Flag-AGO2 and IFNβ reporter were introduced into the 293 cells. Subsequently, IFNβ promoter luciferase activities were measured. ∗*p* < 0.05, ∗∗*p* < 0.01, and ns (no significance), analyzed using a two-tailed Student’s t test; comparisons not marked with bars are with the IgG-IP control. Error bars indicate mean ± SD (*n* = 3). (C) *In vitro* RNA pull-down analysis of AGO2 competition with RIG-I for the binding of biotinylated 5′ppp-RNAs. 293T cells were transfected with the same amount of Flag-RIG-I, while the amounts of V5-AGO2 transfected were ramped up. Biotinylated-5′ppp-RNA was then added to the cell lysate for immunoprecipitation, and the level of RIG-I bound to biotinylated-5′ppp-RNA with increasing amounts of exogenous AGO2 was tested. Left: streptavidin immunoprecipitation; right: input western blot analysis.

To test if AGO2 competes with RIG-I for viral RNA binding, we first obtained viral RNAs associated with RIG-I in DelNS1 WSN influenza virus-infected control (*AGO2*^*WT*^) 293 cells, *AGO2*^−/−^ knockout cells, or *AGO2*^−/−^ knockout cells reconstituted with an exogenous Flag-tagged AGO2 protein. Purified viral RNAs were used for quantitative RT-PCR (Figures S7A and S7B) or to transfect 293 cells for the evaluation of the activation of the IFNβ reporter gene (Figure 6A). Our RT-PCR data reveal a qualitative effect of AGO2 presence on the binding of viral genomic RNA (carrying 5′ppp) to RIG-I. Compared to the control (*AGO2*^*WT*^) and *AGO2*^−/−^ cells overexpressing Flag-AGO2, RNAs co-purified with RIG-I from *AGO2*^−/−^ cells induced IFNβ activation much more effectively (Figure 6A, right). Furthermore, we executed an additional RIG-I CLIP assay to substantiate our findings that AGO2 sequesters viral 5′ppp RNAs bound to RIG-I. To evaluate the direct inhibitory impact of AGO2 on the activation of IFNβ reporter genes, we utilized viral RNAs isolated by co-precipitation with a RIG-I-specific antibody from control (*AGO2* WT) and *AGO2*^−/−^ 293 cells infected with the DelNS1 WSN virus. In this assay, RNA from control and *AGO2* knockout infected cell lines was transfected. The RNA had undergone immunoprecipitation by RIG-I antibody, leading to an enrichment of genomic viral RNA bearing a 5′ppp. We also introduced doses of Flag-AGO2 into the cells in a stepwise manner. We observed that as the concentration of exogenous AGO2 increased, it began to sequester the 5′ppp RNA previously immunoprecipitated by RIG-I. This sequestration process, in turn, modulated the activity of the IFNβ reporter. The results showed that, in comparison to control (*AGO2*^*WT*^) cells, RNAs co-purified with RIG-I from *AGO2*^−/−^ cells induced higher levels of IFNβ activation, and that the activation of IFNβ reporter activity was significantly inhibited by the ectopic expression of Flag-AGO2 in a dosage-dependent manner (Figure 6B). Finally, to further characterize the mechanism by which AGO2 competes to prevent RIG-I from effectively sensing viral RNA, an *in vitro* biotinylated RNA pull-down assay was performed. Biotinylated RNAs bearing 5′-triphosphates and hairpin structure^47^^,^^48^^,^^49^ were used to pull down RIG-I from cells co-transfected with plasmids encoding Flag-RIG-I and an increasing amount of the target protein V5-AGO2 or control protein (GFP). Immunoblotting analysis demonstrated that the interaction between RIG-I and biotinylated 5′ppp-RNA decreased with an increasing amount of AGO2, but not with control GFP (Figure 6C).

**Figure 7:**
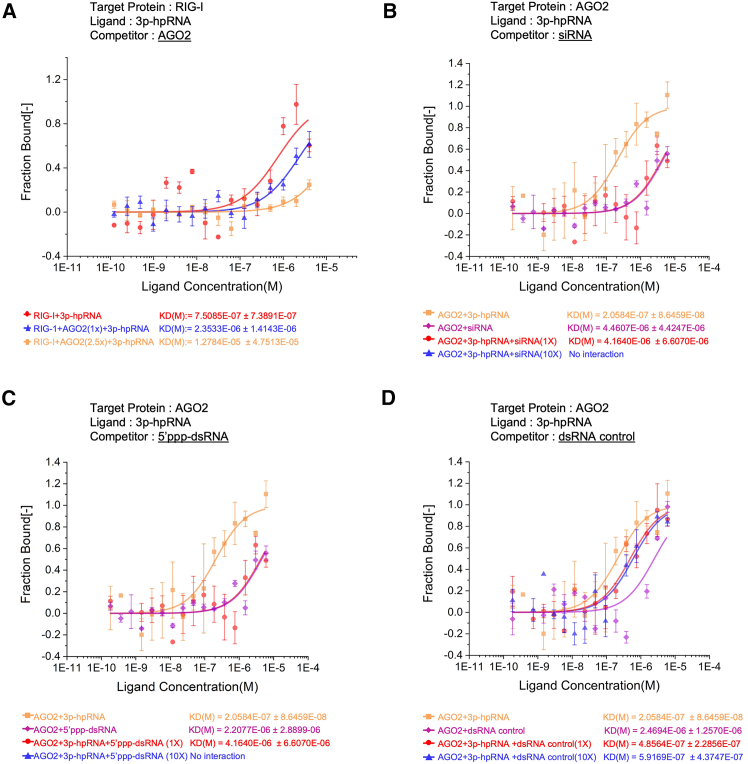
AGO2 binds 5′ppp hairpin RNA directly and competes with RIG-I (A) MST binding assay. The affinity of 3p-hpRNA to RIG-I was measured in the presence of increasing concentrations of AGO2 as a competitor. (B–D) The affinity of 3p-hpRNA to AGO2 was assessed using different competitors (siRNA, 5′ppp-dsRNA, and dsRNA control) at two concentrations (1X and 10X). Binding affinity is plotted as the fraction bound (0 = unbound, 1 = bound) against ligand concentrations ranging from 1 E^−10^ to 1 E^−5^ M. The Kd values, determined by fitting the data as described in method details, are displayed below each graph.

To confirm that 5′ppp hairpin RNA directly binds AGO2 and competes with RIG-I, we employed Microscale Thermophoresis (MST), a precise technique for measuring molecular binding affinities in solution. Our results showed that AGO2 competes with RIG-I for binding to 5′ppp hairpin RNA (3p-hpRNA) in a dose-dependent manner (Figure 7A; Figure S8A). Importantly, this interaction was direct and not mediated by miRNAs, as the assay was conducted in a minimal system containing only AGO2, RIG-I, and 3p-hpRNA. This finding reinforces our proposed model and addresses potential concerns regarding non-specific interactions in cellular overexpression experiments.

Additionally, we used MST binding assays to directly compare the affinity of AGO2 for 5′ triphosphate hairpin RNA (3p-hpRNA) against other RNA ligands, including siRNA, 5′ppp-dsRNA, and a dsRNA control. The results showed that AGO2 binds to 3p-hpRNA with higher affinity (Kd = 0.205 μM) compared to siRNA (Kd = 4.46 μM), 5′ppp-dsRNA (Kd = 2.20 μM), and the dsRNA control (Kd = 2.20 μM). Introducing 1x concentrations of siRNA or 5′ppp-dsRNA partially reduced 3p-hpRNA binding to AGO2, while the excessive loading of these competitors (10x) completely disrupted the interaction between AGO2 and 3p-hpRNA (Figures 7B and 7C; Figure S8B). This suggests that siRNA and 5′ppp-dsRNA may share the same binding site on AGO2. In contrast, the dsRNA control, which lacks the 5′ triphosphate moiety, did not specifically interfere with the AGO2–3p-hpRNA interaction, as even a 10x concentration of the dsRNA control failed to disrupt the binding between AGO2 and 3p-hpRNA (Figure 7D; Figure S8B). These findings further clarify the specificity and competitive binding dynamics of AGO2 for 5′ triphosphate RNA ligands. Collectively, our results provide evidence for a mechanism in which AGO2 directly competes with RIG-I for binding to viral 5′ppp RNAs, thus reducing RIG-I’s ability to recognize and bind to these viral RNAs. This competition ultimately inhibits the activation of the RIG-I-mediated signaling pathway, which is crucial for mounting an effective antiviral immune response.

## Discussion

AGO2, also known as Argonaute 2, is a key component of the RNA-induced silencing complex (RISC), which is involved in the regulation of gene expression through RNA interference (RNAi) and microRNA (miRNA)-mediated silencing. Apart from its role in RNAi, our study reveals AGO2 can modulate RIG-I-mediated antiviral immunity through its ligand interactions.

Effective sensing of non-self RNA and rapid activation of RIG-I signaling play a pivotal role in triggering innate immunity in response to virus infection.^50^^,^^51^ These processes are tightly regulated by several mechanisms affecting the RIG-I protein at the post-translational, post-transcriptional, and functional levels, which are mediated by cellular host proteins or regulatory non-coding RNAs.^13^ Such intricate and tight controls in networks of these modulators of RIG-I signaling enable the initiation of a robust and timely response to virus infection and maintain host immune surveillance. One study has characterized the involvement of AGO2 in attenuating RIG-I signaling to occur through competition with the IRF3-CBP interaction to inhibit the transcriptional expression of antiviral genes, but not by suppressing the phosphorylation of IRF3 or the stability and DNA affinity of IRF3.^20^ Intriguingly, we found that the deficiency of AGO2 dramatically elevated the levels of IRF3 phosphorylation.

RIG-I and MDA5 are both pattern recognition receptors (PRRs) that recognize viral RNA and play crucial roles in the innate immune response. We found that ectopically expressed AGO2 selectively influenced the activation of IRF3 and IFNβ and promoter-driven luciferase in a dose-dependent manner in response to RIG-I ligands, but not to MDA5 ligands (Figures 3B and 3C). The RIG-I ligands (3p-hpRNA, poly(A:T), and poly(I:C)-LMW) are characterized by the presence of a 5′-triphosphate group and a double-stranded RNA (dsRNA) structure,^23^^,^^25^ which are recognized by the helicase domain and the C-terminal domain (CTD) of RIG-I. On the other hand, MDA5 ligands such as high molecular weight poly(I:C) (poly(I:C)-HMW) are long dsRNAs that are preferentially recognized by MDA5 due to its ability to oligomerize along the dsRNA.^23^^,^^25^^,^^26^^,^^52^^,^^53^ The exact mechanism behind AGO2’s preference for RIG-I ligands is not fully understood, but it is likely related to the structural preferences of AGO2 and its specific recognition sites. AGO2 may have a higher affinity for the 5′-triphosphate group or the specific hairpin structures found in RIG-I ligands, while it may not efficiently interact with the longer dsRNA structures preferred by MDA5. The 3′ and 5′ untranslated regions (UTRs) of influenza vRNA segments are well-documented to form panhandle structures, which are structurally analogous to double-stranded hairpins. These structures play critical roles in various aspects of the viral life cycle, including RNA replication, transcription, and packaging. Additionally, influenza vRNA carries a 5′ triphosphate (5′ppp) structure, which, together with the panhandle, is known to directly activate RIG-I, thereby inducing antiviral responses.^54^ Conversely, influenza A virus-derived small viral RNAs (svRNAs) with 22–27 nt in length, even in the presence of 5′-triphosphates, failed to induce an IFN response.^55^ Additionally, it is possible that the presence of specific cellular co-factors or post-translational modifications could modulate AGO2’s binding preferences, leading to a selective interaction with RIG-I ligands. To further investigate this hypothesis, it would be important to perform experiments to identify the specific interacting domains, characterize the nature of their interaction, and determine how their interaction affects the RNA-binding properties and functions of both proteins.

AGO2 is a multidomain protein, consisting of an N-terminal domain, PAZ domain, MID domain, and PIWI domain. The PAZ domain is known to bind the 3′ end of small RNAs, whereas the MID domain interacts with the 5′ end. The PIWI domain is structurally similar to RNase H and is responsible for the endonucleolytic cleavage of target RNAs.^56^^,^^57^^,^^58^ Our study provides further insights into the molecular mechanisms by which AGO2 modulates RIG-I-mediated antiviral immunity through domain mapping analysis. The observed co-precipitation of RIG-I and AGO2 in mock-infected cells suggests a potential interaction between these two proteins in the absence of viral infection. However, the decrease in their association following DelNS1 WSN or SeV infection, both of which lead to viral RNA production, implies that the presence of viral RNA might hinder the binding between RIG-I and AGO2. It is noteworthy that RIG-I can also be ubiquitinated upon binding to 5′ppp RNA at the CTD (C-terminal domain), inducing further conformational changes that result in the formation of a highly active signaling tetramer.^29^^,^^59^ This tetrameric form of RIG-I is crucial for the efficient activation of downstream signaling pathways, such as IRF3. It is possible that when RIG-I binds to viral RNA, it undergoes a conformational change that exposes its CARDs, enabling interaction with the mitochondrial antiviral-signaling protein (MAVS).^60^ Conversely, this binding may hinder the interaction with AGO2. Furthermore, structural mapping analysis demonstrated that only the N-terminal and PIWI domains of AGO2 efficiently immunoprecipitate RIG-I protein, suggesting that these domains play a crucial role in the interaction between the two proteins. In contrast, all domains of RIG-I except for the CTD were found to bind to AGO2. Interestingly, the C-terminal domain of RIG-I is responsible for recognizing both double-stranded RNA (dsRNA) and 5′ppp single-stranded RNA (ssRNA),^23^^,^^25^^,^^29^^,^^44^^,^^45^^,^^46^ while it is the MID and PAZ domains of AGO2 that bind small RNA molecules,^61^^,^^62^^,^^63^^,^^64^ with the MID domain interacting with the 5′ end of the small RNA and helping anchor the 5′ phosphate of the RNA^46^^,^^61^ and the PAZ domain specifically binding to the 3′ end of small RNA molecules.^65^ Our MST binding assays directly compared the affinity of AGO2 for 5′ monophosphate siRNA versus 5′ triphosphate hairpin RNA (3p-hpRNA) (Figure 7B; Figure S8B). The results demonstrated that AGO2 binds 3p-hpRNA with higher affinity than siRNA. Moreover, excessive siRNA (10x) completely disrupted the interaction between AGO2 and 3p-hpRNA, suggesting that these RNAs may compete for the same binding site on AGO2, likely involving the MID domain.

Our findings indicate that the RNA binding domains of both proteins, namely the CTD of RIG-I and the PAZ and MID domains of AGO2, do not directly interact with each other. Intriguingly, this interaction is diminished in the presence of viral RNA during delNS1 and Sev infections (Figure 5A), suggesting that the RNA-binding pocket of AGO2 is essential for 5′ triphosphate RNA binding but not for its interaction with RIG-I. These observations raise the question of how these proteins regulate each other’s function in the context of antiviral immunity. One possibility is that AGO2 and RIG-I compete for binding to viral RNA through their respective RNA-binding domains, with AGO2 acting as a decoy to sequester viral RNA away from RIG-I. Alternatively, the interaction between AGO2 and RIG-I could modulate the conformation or activity of either protein, thereby affecting their ability to bind and process viral RNA. Moreover, specific interactions between the N-terminal and PIWI domains of AGO2 and the CARD and helicase domains of RIG-I may play a crucial role in regulating downstream signaling events in RIG-I-mediated antiviral pathway. For example, the interaction between AGO2 and the CARD domain of RIG-I could interfere with the activation of downstream signaling components, such as MAVS, and thus attenuate the antiviral response. Our findings highlight the complexity of the interplay between AGO2 and RIG-I and their roles in modulating antiviral immunity. Understanding the molecular details of these protein-protein interactions and their functional consequences will be critical for elucidating the mechanisms by which viruses evade host immune defenses and for developing novel antiviral therapies. Further studies are needed to determine the precise molecular basis for the observed domain-specific interactions between AGO2 and RIG-I and to explore how these interactions impact on antiviral immunity.

In summary, AGO2 modulates RIG-I-mediated antiviral immunity through its structural features and ligand interactions. The ability of AGO2 to interact with RIG-I and bind viral RNA, highlights the complexity of the host-virus interplay in the regulation of antiviral immunity. Further studies are needed to fully understand the role of AGO2 and other host proteins in the immune response to viral infections and to develop novel therapeutic strategies targeting these proteins to enhance antiviral defense.

### Limitations of the study

While our research sheds light on AGO2’s function in RIG-I-mediated immune responses, several limitations exist. Most of our data are derived from *in vitro* and overexpression systems, highlighting the need for validation in primary cells and animal models. The domain-mapping experiments do not fully elucidate the mechanistic impact of these interactions on signaling pathways, particularly since post-translational modifications of AGO2 and RIG-I were not thoroughly examined. Additionally, the potential roles of other host factors involved remain unclear. The complex role of RNAi during viral infections was not addressed in this study, emphasizing the need for future research into its specific functions against influenza A and other viruses.

## Resource availability

### Lead contact

Further information and requests for resources and reagents should be directed to and will be fulfilled by the lead contact, Bobo Mok (bobomok@hku.hk).

### Materials availability

All unique cells and plasmids generated and described in this study are available from the lead contact upon completion of a material transfer agreement after request by qualified academic investigators for non-commercial purposes.

### Data and code availability

• **Data:** Data reported in this paper will be shared by the lead contact upon request.
• **Code:** This paper does not report original code.
• **Additional information:** Any additional information required to reanalyze the data reported in this paper is available from the lead contact upon request.

## Acknowledgments

The authors appreciate Dr. Chul Kwon for his insightful information and valuable discussions. We also thank Zoonbio Biotechnology Co. Ltd. for conducting the MST experiment. Acknowledgment is also extended to Dr. Jane Rayner for her careful reading and editing of the article. This study is partly supported by Health@InnoHK, Innovation and Technology Commission, the Theme-Based Research Scheme (T11-709/21-N), the Collaborative Research Fund (C5110-20GF), and the General Research Fund (17107019) of the Research Grants Council, the 10.13039/501100005847Health and Medical Research Fund Commissioned Research on COVID-19 (COVID1903010, COVID190123) and 10.13039/501100005847HMRF (19181052 and 14131032), Hong Kong, the Tier 1 Research Start-up Grants from the Research Committee of Hong Kong Baptist University (162874), the Hong Kong Special Administrative Region, China, the Emergency Collaborative Project (EKPG22-01) of Guangzhou Laboratory, China, and the Shenzhen Science and Technology Program (JCYJ20240813145001002). Graphical abstract was created in BioRender. CVVT, C. (2025) https://BioRender.com/gcddatc.

## Author contributions

Conceptualization: H.L. and B.W.M. Methodology: H.L., Y.L., F.Y., L.N.L., Y.Z., L.Z., G.X., J.L., S.L., S.D., R.C.T., W.S., P.C., X.H., C.J.C., M.Z., P.W., and B.W.M. Software: H.L., F.Y., L.Z., G.X., and B.W.M. Validation: H.L. and B.W.M. Formal analysis: H.L. and B.W.M. Investigation: H.L. and B.W.M. Resources: Z.C., K.Y., and H.C. Supervision: H.C. and B.W.M. Data Curation: H.L. and B.W.M. Visualization: H.L. and B.W.M. Writing - Original Draft: H.L., H.C., and B.W.M. Writing - review and editing: H.L., H.C., and B.W.M. Project administration: B.W.M. Funding acquisition: P.W., L.Z., Z.C., K.Y., H.C., and B.W.M.

## Declaration of interests

The authors declare no competing interests.

## STAR★Methods

### Key resources table

**Table:** 

REAGENT or RESOURCE	SOURCE	IDENTIFIER
**Antibodies**		

Rat monoclonal anti-AGO2 (Clone 11A9)	Merck Millipore	Cat# MABE253, RRID:AB_2938521
Rabbit polyclonal anti-AGO2	Abcam	Cat# ab32381, RRID:AB_867543
Mouse monoclonal anti-DICER (13D6)	Abcam Or Santa Cruz Biotechnology	Cat# ab14601, RRID:AB_443067
Rabbit monoclonal anti-DROSHA (D28B1)	Cell Signaling Technology	Cat# 3364, RRID:AB_2238644
Rabbit polyclonal anti-RIG-I	ABclonal	Cat# A0550, RRID:AB_2757259
Mouse monoclonal anti-RIG-I (Alme-1)	AdipoGen Life Sciences	Cat# AG-20B-0009, RRID:AB_2490189
Mouse monoclonal anti-Stat1	ABclonal	Cat# A10100, RRID:AB_2757623
Rabbit monoclonal anti-Phospho-Stat1 (Tyr701)	Cell Signaling Technology	Cat# 9167, RRID:AB_561284
Mouse monoclonal anti-MAVS (E−3)	Santa Cruz Biotechnology	Cat# sc-166583, RRID:AB_2012300
Mouse monoclonal anti-IRF3	Santa Cruz Biotechnology	Cat# sc-33641, RRID:AB_627826
Rabbit monoclonal anti-Phospho-IRF3(Ser386)	Abcam	Cat# ab76493, RRID:AB_1523836
Rabbit monoclonal anti-Influenza virus NS1	A gift from Dr. Tan Yee Joo, National University of Singapore	N/A
Mouse monoclonal anti-Influenza virus M1 (GA2B)	Santa Cruz Biotechnology	Cat# sc-57881, RRID:AB_784085
Mouse monoclonal anti-Influenza virus NP	A gift from Xiamen University, China	N/A
Mouse monoclonal anti-Influenza virus PB1	A gift from Xiamen University, China	N/A
Mouse monoclonal anti-Influenza virus PB2	A gift from Xiamen University, China	N/A
Mouse monoclonal anti-Influenza virus PA	A gift from Xiamen University, China	N/A
Rabbit polyclonal anti-alpha Tubulin	Abcam	Cat# ab18251, RRID:AB_2210057
Mouse monoclonal anti-beta Actin	Abcam	Cat# ab8226, RRID:AB_306371
Rabbit polyclonal anti-Flag	Thermo Fisher Scientific	Cat# PA1-984B, RRID:AB_347227
Mouse monoclonal anti-Flag	Sigma-Aldrich	Cat# F1804, RRID:AB_262044
Mouse monoclonal anti-V5 (clone SV5-Pk1)	Bio-Rad	Cat# MCA1360, RRID:AB_322378)
Rabbit monoclonal anti-V5 (clone D3H8Q)	Cell Signaling Technology	Cat# 13202, RRID:AB_2687461
Rabbit polyclonal anti-GFP	Sigma-Aldrich	Cat# SAB4301138, RRID:AB_2750576

**Bacterial and virus strains**		

A/WSN/1933 (H1N1) (WSN)	lab strain (Zheng et al.^68^)	N/A
DelNS1 WSN (ΔNS1 WSN)	lab strain (Zheng et al.^68^)	N/A
SeV	Professor Dongyan Jin, The University of Hong Kong	N/A

**Chemicals, peptides, and recombinant proteins**		

Puromycin	Gibco	A1113803
Proteinase K	Thermo Fisher Scientific	EO0492
RNA 5′ Pyrophosphohydrolase (RppH)	New Enland Biolabs	M0356S
RNAse A/T1 Mix	Thermo Fisher Scientific	EN0551
RNaseOUT Recombinant Ribonuclease Inhibitor	Thermo Fisher Scientific	10777019
Lipofectaimine RNAiMAX	Invitrogen	13778075
Lipofectamine 3000	Invitrogen	L3000015
Polyethylenimine	Polysciences Inc	23966
Dithiothreitol (DTT)	MedChemExpress	HY-15917
Turbo DNase I	Thermo Fisher Scientific	AM2238
3p-hpRNA (5′ppp hairpin RNA)	InvivoGen	tlrl-hprna
Poly(A:T)	InvivoGen	tlrl-patn
Poly(I:C) LMW	InvivoGen	tlrl-picw
Poly(I:C) LMW	InvivoGen	tlrl-pic
5′ppp-dsRNA	InvivoGen	tlrl-3prna
5′ppp-dsRNA Control	InvivoGen	tlrl-3prnaclv

**Critical commercial assays**		

MEGAscript T7 Transcription Kit	Thermo Fisher Scientific	AMB13345
Dual-Luciferase Reporter Assay System	Promega	E1910
Quick -RNA Microprep Kit	Zymo Research	R1051
PrimeScript RT Reagent Kit	Takara	RR037A
SYBR Green Premix Ex Taq Kit	Takara	RR420A
TaqMan™ MicroRNA Reverse Transcription Kit,	Thermo Fisher Scientific	4366596
TaqMan® MicroRNA Assays	Thermo Fisher Scientific	4427975, 000391, hsa-miR-16, 002277, has-miR-320, 001973, U6 snRNA
TaqMan™ Universal Master Mix II, no UNG	Thermo Fisher Scientific	4440043
Monolith Protein Labeling Kit RED-NHS 2ND Generation Kit	NanoTemper	MO-L011

**Experimental models: Cell lines**		

Human lung carcinoma cell line A549	ATCC	Cat# CCL-185
Human embryonic kidney cell line HEK293	ATCC	Cat# CCL-1573
Human embryonic kidney cell line HEK293T	ATCC	Cat# CRL-11268
Madin-Darby Canine Kidney MDCK	ATCC	Cat# CCL-34
A549-Control	This paper	N/A
A549-AGO2-KO	This paper	N/A
293-Control	This paper	N/A
293-AGO2-KO	This paper	N/A
293-AGO2/DROSHA-KO	This paper	N/A
A549-DICER-KO	This paper	N/A
A549-DROSHA-KO	This paper	N/A
293 scramble	Schmitter et al.^67^	N/A
293 AGO2-kd	Schmitter et al.^67^	N/A
293T NoDice cells	Bogerd et al.^41^	N/A
293T Parental cells	Bogerd et al.^41^	N/A

**Oligonucleotides**		

Uni-12,see Table S1	This paper	N/A
WSN NP^512nt^ (*in vitro* pull down assay), see Table S1	This paper	N/A
gRNA sequence for generating KO clones, see Table S1	This paper	N/A
Primers for PCR and CHIP, see Table S1	This paper	N/A
siRNA (5′ *p*-GCAUGCGACCU CUGUUUGA-dTdT-3′)	This paper	N/A

**Recombinant DNA**		

Flag-AGO2	This paper	N/A
V5-AGO2	This paper	N/A
V5-AGO2-N-1∼139aa	This paper	N/A
V5-AGO2-N-L-1∼229aa	This paper	N/A
V5-AGO2-PAZ-L2-230∼445aa	This paper	N/A
V5-AGO2-MID-446∼580aa	This paper	N/A
V5-AGO2-PIWI-581∼859aa	This paper	N/A
Flag-RIG-I	This paper	N/A
Flag-RIG-I-ΔCARD-231∼925aa	This paper	N/A
Flag-RIG-I-ΔCTD-1∼790aa	This paper	N/A
Flag-RIG-I- CARD-1∼230aa	This paper	N/A
Flag-RIG-I-Helicase-231∼790aa	This paper	N/A
Flag-RIG-I-CTD-791∼925aa	This paper	N/A
Flag-MAVS	This paper	N/A
V5-IRF3-5D	This paper	N/A
Flag-TBK1	This paper	N/A
Flag-IKKε	This paper	N/A
IFN-β-luciferase-reporter	Professor Dongyan Jin, The University of Hong Kong	N/A
phRL-TK (Renilla luciferase)	This paper	N/A
pX459 CRISPR/Cas9-Puro vector	Laboratory of Feng Zhang	Addgene # 48139; RRID: Addgene_48139
pSpCas9(BB)-2A-Puro (PX459)-AGO2-gRNA	This paper	N/A
pSpCas9(BB)-2A-Puro (PX459)-DICER-gRNA	This paper	N/A
pSpCas9(BB)-2A-Puro (PX459)-DROSHA-gRNA	This paper	N/A
BII-Bla-tdTomato	Professor SC Kwon, The University of Hong Kong	N/A
pLKO-puro (control)	Professor SC Kwon, The University of Hong Kong	N/A
pLKO-shTomato4 (knocking down Tomato)	Professor SC Kwon, The University of Hong Kong	N/A
pU6 (control)	Professor SC Kwon, The University of Hong Kong	N/A
pU6-agoshTomato6 (knocking down Tomato)	Professor SC Kwon, The University of Hong Kong	N/A

**Software and algorithms**		

GraphPad Prism software	GraphPad Software, Inc, San Diego, CA,USA	version 9
AzureSpot Pro analysis software	Azure Biosystems	version 1.4-583
OriginPro software	OriginLab, Northampton, MA, USA	2024
Inference of CRISPR Edits (ICE)	Synthego	ICE v3

### Experimental model and study participant details

Human lung carcinoma cell line A549 (ATCC), human embryonic kidney cell line HEK293 (ATCC), human embryonic kidney cell line HEK293T (ATCC) and Madin-Darby Canine Kidney (MDCK-ATCC) cells were cultured in Dulbecco’s Modified Eagle Medium (DMEM) or Minimum Essential Medium (MEM) supplemented with 10% FBS and 1% Penicillin-Streptomycin (P/S) at 37 °C with 5% CO_2_. Doxycycline (Dox) induction of AGO2 knockdown in the 293 cell line (gift from Professor Petr Svoboda, Institute of Molecular Genetics of the Czech Academy of Sciences)^66^ was performed at 5 mg/mL for at least 72 h.

### Method details

#### Viruses and infection

Sendai virus (SeV), a kind gift from Professor Dongyan Jin, Department of Biochemistry, HKU, was grown in embryonated chicken eggs. Influenza virus strains A/WSN/1933 (H1N1) (WSN) and DelNS1 WSN (ΔNS1 WSN) were rescued using the reverse genetics technique in 293T cells and propagated in MDCK cells, as described previously. Generation of DelNS1 WSN was conducted in a previous study by our group.^67^ Confluent 293 or A549 cells were infected with WSN, DelNS1 WSN or SeV virus at different MOI (multiplicity of infection). Infection conditions, including duration and MOI, were optimized for different experiments throughout the study as demonstrated in Figures S9A–S9C. For protein or RNA analysis, infected cells were collected and lysed in cell lysis buffer (50 mM Tris-HCl (pH 7.4), 150 mM NaCl, 1% Triton X-100, and protease inhibitor cocktail (Roche)) or RNAzol RT (Molecular Research Center, Inc) at the indicated time points.

#### Plasmid construction and mutagenesis

Expression constructs for AGO2 with a Flag or V5 tag were cloned into pCDNA3.1 vector. Expression constructs for RIG-I, 2CARD, TBK1, IRF3 and IRF3-5D were constructed in the pEF-BOS vector with Flag or V5 tags at their N-terminal or C-terminal. Expression constructs for AGO2 truncations or other types of mutants, and RIG-I truncations were generated using Q5 High-Fidelity DNA Polymerase (NEB). These constructs were generated by ligation-independent cloning involving exonuclease III digestion, as described previously. For generation of gene editing vectors by CRISPR-Cas9, gRNA sequences were designed and cloned into vector pSpCas9 (BB)-2A-Puro (PX459) V2.0 (Addgene) provided by Professor Zhang Feng.^68^

#### CRISPR knockout

For gene editing, CRISPR/Cas9 KO plasmids for AGO2, DICER, DROSHA, and were transfected using Lipofectamine 3000 (Thermo Fisher Scientific) into 293 and A549 cells seeded with 50% confluency one day beforehand. After 24 h of transfection, cells were supplemented with fresh medium containing 2–8 mg/mL puromycin (Sigma) and cultured for a further 2 days for selection. Positive single-cell clones of these CRISPR cells were seeded into 96-well plates, then expanded and confirmed at protein and genetic levels. Control cells (*AGO2*^*WT*^) underwent the same procedure as the KO cells. All KO clones utilized in this study have undergone genomic and functional validation (Figures S10A–S10C). For genomic validation, the target regions (sgRNA sites) of each KO clone were amplified by PCR using primers flanking the sgRNA sites (primer sequences are listed in Table S1). The resulting PCR products were subsequently subjected to Sanger sequencing. The sequencing data were analyzed using the Inference of CRISPR Edits (ICE) tool (Synthego) to confirm the presence and nature of the intended genetic modifications. This comprehensive validation ensures the reliability and accuracy of the KO clones used in our experiments.

#### Nucleic acid preparation and enzymatic treatment

Influenza virus WSN genomic RNAs were extracted from purified viral particles using TRIzol LS (Life Technologies) according to the manufacturer’s manual. Transcripts of gene segments of influenza virus were generated by *in vitro* transcription (IVT) in which the templates were amplified from plasmids with the relevant coding regions using PrimeSTAR GXL DNA Polymerase (Takara) and transcribed RNAs were obtained using a MEGAscript T7 Transcription Kit (Thermo Fisher Scientific). For enzymatic treatment, RNA 5′ Pyrophosphohydrolase (*RppH*) was used to remove pyrophosphate from the 5′ end of triphosphorylated RNA according to the manufacturer’s manual (NEB).

#### Cell stimulation and luciferase reporter assay

To evaluate the effects of AGO2 on IFNb signaling, 293 cells were transfected with 3p-hpRNA (1 mg/mL, InvivoGen, tlrl-hprna), poly(A:T) (1 mg/mL, InvivoGen, tlrl-patn), short poly(I:C) (LMW) (2 mg/mL, InvivoGen, tlrl-picw), and long poly(I:C) (HMW) (2 mg/mL, InvivoGen, tlrl-pic) using polyethylenimine (PEI; Polysciences Inc.) or Lipofectamine 3000. At the indicated times, cells were collected and lysed in RNAzol RT for further study. To determine the effect of AGO2 on IFNb or IRF3 activity, 293 cells were transfected with an IFNβ or IRF3 promoter-driven luciferase reporter, a control renilla luciferase reporter and an increasing dose of the plasmid encoding Flag-AGO2 for 10 h. After refreshing the medium, cells were transiently transfected with different stimuli including 3p-hpRNA, poly(A:T), short poly(I:C) (LMW) and poly(I:C) (HMW) or RIG-I purified viral RNAs using PEI for 16–24 h, and then collected in passive lysis buffer to evaluate In another assay to detect IFNβ activities, IFNb luciferase reporter and renilla luciferase reporter, together with other inducers including viral RNA, AT-rich dsRNA (poly(A:T)), 5′ppp-RNA (InvivoGen, tlrl-3prna) or IVT RNA, or plasmids encoding RIG-I-2CARD, TBK1, IRF3, or IRF3-5D, or RNA species precipitated by AGO2, were co-transfected into 293 cells seeded in 48-well plates. After 16–24 h of incubation, luciferase activities were performed using the Dual-Luciferase Reporter Assay System according to the manufacturer’s manual. Firefly luciferase activity was calculated by normalization to the activity of renilla luciferase.

#### Immunoblotting

Cells were lysed with lysis buffer (150 mM NaCl, 50 mM Tris, pH 7.4, 1% Triton X-100, 1 mM EDTA with a protease inhibitor cocktail (cOmplete EDTA-free, Roche) and phosphatase inhibitor cocktail (PhosSTOP, Sigma)) for 30–60 min on ice. After centrifugation, supernatants were mixed with 6 x sample buffer (0.35 M Tris-HCl pH 6.8, 10% SDS, 30% glycerol, 0.6 M DTT and 0.12% bromophenol blue) and boiled at 95 °C for 10 min. Boiled samples were loaded into sodium dodecyl sulfate polyacrylamide gel electrophoresis (SDS-PAGE) with separating gels at 8%, 10% and 12% concentration depending on protein sizes. After separation, proteins were transferred to nitrocellulose membrane (Bio-Rad) and then blocked in 3% skim milk (Sigma) in PBS for at least 30 min. Membranes were incubated with the following primary antibodies used at 1:1000-1:5000 dilution in blocking buffer (2.5% BSA and 0.05% NaN3 in PBS) overnight at 4 °C: rat-AGO2 (Merck Millipore, Clone 11A9), rabbit-AGO2 (Abcam, ab32381), mouse-DICER (Santa Cruz Biotechnology or Abcam, ab14601), rabbit-DROSHA (Cell Signaling Technology), rabbit-RIG-I (ABclonal, A0550), mouse-RIG-I (AdipoGen Life Sciences, Alme-1), mouse-STAT1 (ABconal, A10100), rabbit-Phospho-STAT1 (Tyr701) (Cell Signaling Technology, 58D6), mouse-MAVS (Santa Cruz Biotechnology, E−3), mouse-IRF3 (Santa Cruz Biotechnology, SL-12), rabbit-Phospho-IRF3 (Abcam, ab76493), rabbit-Influenza virus NS1 (A gift from Dr. Tan Yee Joo, National University of Singapore), mouse-Influenza virus M1 (Santa Cruz Biotechnology, GA2B), mouse-Influenza virus NP, PB1 and PB2 (gifts from Xiamen University, China), rabbit a-TUBULIN (Abcam, ab18251), mouse b-ACTIN (Abcam, ab8226), rabbit-Flag (Thermo Fisher Scientific, PA1-984B), mouse-Flag (Sigma, M2/F1804), mouse-V5 (Bio-Rad, clone SV5-Pk1), rabbit-V5 (Cell Signaling Technology, clone D3H8Q), rabbit-GFP (Sigma). Membranes were then incubated with secondary antibody (IRDye-680 or -800, LI-COR Biosciences) diluted in PBST for 1 h at room temperature. After washing with 0.05% PBST and PBS, membranes were dried, scanned with an Odyssey imaging system (LI-COR Biosciences), and analyzed with ImageJ (NIH).

#### Co-immunoprecipitation

For co-immunoprecipitation assays using an overexpression system, 293 cells were seeded in a 6-well plate one day prior to transfection with the indicated protein expression plasmids. After 48 h incubation, the cells were either infected with virus or mock-infected, and subsequently harvested. For the endogenous protein co-immunoprecipitation assay, 293 cells or A549 cells were seeded in a 10-cm dish one day prior to being infected with DelNS1 WSN or SeV or mock-infected. Harvested cells were washed twice with cold PBS, lysed at 4 °C for 30–60 min in low salt lysis buffer (150 mM NaCl, 50 mM Tris, pH 7.4, 1% Triton X-100, 1 mM EDTA with a protease inhibitor cocktail and a phosphatase inhibitor cocktail). The lysates were centrifuged at 12000 g for 10 min 5% of each supernatant sample was kept as sample input. The remaining supernatant was incubated with anti-Flag M2 magnetic beads (Sigma) at 4 °C overnight or with the corresponding antibody previously incubated with Dynabeads Protein A or G (Thermo Fisher Scientific) at 4 °C overnight. Antibodies used in this assay were: mouse-Flag (Sigma, M2/F1804), mouse-V5 (Bio-Rad, clone SV5-Pk1), rat-AGO2 (Merck Millipore, Clone 11A9), rabbit-RIG-I (ABclonal, A0550), control IgG antibodies (Santa Cruz Biotechnology). Subsequently, the beads were washed five times with ice-cold lysis buffer. The precipitated proteins were then eluted using 2x SDS sample buffer, denatured at 95°C for 10 min, and subjected to western blot analysis as described above.

#### Quantitative RT-PCR

Total RNAs from cells were extracted using RNAzol RT according to the manufacturer’s manual. RNAs were reverse-transcribed into complementary DNA (cDNA) using the PrimeScript RT Reagent Kit (Takara) according to the manufacturer’s manual. For host gene and viral messenger RNA (mRNA) detection, oligo (dT) primers were used while universal 12 was used for viral genomic RNA (vRNA). cDNAs were diluted 20-fold with ultra-pure water. Quantitative reverse transcription PCR (RT-qPCR) was then performed using SYBR Green Premix Ex Taq Kit (Takara) according to the manufacturer’s manual and gene expression detected in a LightCycler 480 Instrument (Roche). The 2^-ΔΔCT^ was used to calculate relative gene expression. Primers used in this assay are listed in Table S1.

#### Crosslinking and immunoprecipitation (CLIP) of AGO2 or RIG-I associated RNA

Isolation of AGO2 or RIG-I associated RNAs by crosslinking and immunoprecipitation (CLIP) was modified from a previous protocol.^69^ Briefly, A549 or 293 cells, seeded in 6-well plates 1 day before, were infected with WSN or DelNS1 WSN virus at an MOI of 1–2 for 8 h. Cells were irradiated within their medium at 400 mJ/cm^2^ and then 200 mJ/cm^2^ using a Stratalinker 2400 (Stratagene). After washing with cold PBS, cells in each well were lysed in 220 mL lysis buffer (150 mM NaCl, 50 mM Tris, pH 7.4, 1% Triton X-100, 1 mM EDTA with a protease inhibitor cocktail) on ice for 30–60 min and then digested with 10 U of Turbo DNase I (Invitrogen) at 37 °C for 10 min. After centrifugation, 5% of each supernatant sample was retained as sample input and the remaining supernatant combined with an RNase inhibitor (RNaseOUT Recombinant Ribonuclease Inhibitor, Thermo Fisher Scientific) at a final concentration of 0.5 U/mL and incubated overnight with 10 mL Dynabeads Protein G that had been previously incubated with 1 mg of anti-AGO2 antibody (11A9) or 1 mg of anti-RIG-I (A0550) or normal control IgG antibody. Precipitates were then washed five times with ice-cold lysis buffer supplemented with RNAase inhibitor (0.05 U/mL) and subsequently subjected to proteinase K digestion in a buffer containing (85 μL of PBS, 15 μL of 10%SDS and 10 μL of 20 mg/mL Proteinase K). The samples were then processed using column-based RNA extraction kits (Zymo Research). Immunoprecipitated RNA was treated with 500 U/mL of RppH (NEB) at 37°C for 1 h with shaking at 1100 rpm, followed by purification using an RNA extraction column. Following CLIP, purified RNAs were quantified by RT-qPCR or transfected into 293 cells for IFNb luciferase reporter assay.

#### *In vitro* RNA pull-down assay

The biotinylated-5′-ppp-RNA used in our study was generated through *in vitro* transcription (IVT) using T7 polymerase, with templates amplified from a plasmid encoding the influenza WSN nucleoprotein (NP). The 5′-ppp group and the hairpin structure is a result of the IVT process,^47^^,^^48^ both of which are crucial for the activation of RIG-I.^49^ Sequences of primers used for generating 5′ppp-IVT-NP RNA^512nt^ are listed in Table S1. To ensure correct secondary structure of RNAs in pull-down assay, synthesized RNAs were heated at 65 °C for 10 min, put on ice for 2 min and left at room temperature for 20–30 min. To prepare RNA binding proteins, 293 cells in 6-well plates were transfected with V5-AGO2, Flag-RIG-I, GFP or control plasmids using PEI. After 24–30 h of incubation, cells were resuspended in 220 μL lysis buffer (150 mM NaCl, 50 mM Tris, pH 7.4, 1% Triton X-100, 1 mM EDTA with a protease inhibitor cocktail) at 4 °C for 30–60 min. After centrifugation at 12,000*g* for 10 min at 4 °C, the supernatant was supplemented with 0.5 U/mL RNase inhibitor and incubated with 4–5 mg of biotinylated RNAs or control RNAs overnight at 4 °C. For preparing RNA-conjugated beads, 100 mL streptavidin beads (M-280, Thermo Fisher Scientific) resuspended in 1 mL of 1x binding and washing buffer (5 mM Tris-HCl, pH 7.5, 0.5 mM EDTA and 1 M NaCl) were rotated in wells of 6-well plates for 5 min at room temperature. Streptavidin beads were washed twice with 400 mL of buffer A (DEPC-treated 0.1 M NaOH and DEPC-treated 0.05 M NaCl), and then were resuspended twice with 400 mL of buffer B (DEPC-treated 0.1 M NaCl) to remove NaOH. The streptavidin beads were then washed with lysis buffer and incubated with cell lysates previously incubated with biotinylated RNAs or control RNAs overnight. After 4–5 h of incubation at 4 °C, streptavidin beads were washed five times with lysis buffer and boiled in 2 x SDS sample buffer at 95 °C for 10 min. The RNA binding proteins were analyzed by western blot.

#### MicroScale thermophoresis (MST)

MicroScale Thermophoresis (MST) (NanoTemper, Germany) was employed to investigate binding affinicty interactions between RIG and AGO2 to different RNA ligand molecules. In a standard labeled MST experiment, up to 16 dilution gradients can be measured simultaneously. The concentration of the fluorescently labeled molecule (Target) was maintained at 5–150 nM, while the unlabeled molecule (Ligand) was serially diluted as indicated. Target protein (RIG-I or AGO2) were purchased from MCE and were labeled using the Monolith RED-NHS 2nd Generation Protein Labeling Kit (NanoTemper, Germany), following the manufacturer’s protocol. Briefly, 90 μL of the target protein solution was labeled, yielding approximately 450 μL of labeled protein solution with a final concentration of 0.1 μM. The ligand molecules (3p-hpRNA, siRNA, 5′ppp-dsRNA and dsRNA and) were serially diluted, and 10 μL of each dilution was mixed with 10 μL of the labeled target protein at a fixed concentration. The mixture was incubated for 5 -15 min to allow binding. Subsequently, the samples were loaded into glass capillaries (MO-k022) and analyzed using the MST-NT.115 Pico device (NanoTemper).

Prior to the main experiment, a pretest was conducted to assess the labeling efficiency of the target protein and to detect potential protein adsorption or aggregation. The target protein was diluted with assay buffer (containing 0.05% Tween 20) to a working concentration of 5–150 nM. A 15 μL aliquot of the diluted target protein was mixed with 15 μL of assay buffer, and the mixture was loaded into a capillary for analysis using the NT115 Pico device.

If no protein aggregation or adsorption is observed, the binding affinity assay is initiated. Small molecules are dissolved in an aqueous solution, and the small molecule stock solution is serially diluted using a 2-fold concentration gradient across 16 tubes, with each tube containing 10 μL of the diluted solution. Subsequently, 10 μL of the diluted protein solution is added to each tube and thoroughly mixed by gentle pipetting. The mixture is allowed to equilibrate for 5–15 min before being aspirated into a capillary tube for instrument analysis. A signal-to-noise ratio greater than 5 is used as a quality control criterion to confirm binding. This process is repeated to ensure reproducibility and accuracy in determining binding affinity. For binding affinity analysis, ligand-dependent changes in microscale thermophoresis (MST) are plotted as normalized fluorescence (Fnorm) values against ligand concentration to generate a dose-response curve. Fnorm values are expressed in parts per thousand (‰). For each capillary (representing a single measurement point), an MST trace is recorded. All traces are normalized to start at a relative fluorescence value of 1. The Fnorm value for the dose-response curve is calculated by dividing the relative fluorescence in the heated state (F1) by the relative fluorescence in the cold state (F0), typically measured before the IR laser is activated. Fnorm values are typically reported in ‰.The Kd fit model describes a molecular interaction with a 1:1 stoichiometry according to the law of mass action.The Kd is estimated by fitting the equation:

$$f\left(c\right)=Unbound+\left(Bound-Unbound\right)\times \frac{c+c_{target}+K_{d}-\sqrt{{\left(c+\;c_{target}+K_{d}\right)}^{2}-4{\;c\;c}_{target}}}{2\;c_{target}}$$

where f(c) is the fraction bound at a given ligand concentration c; Unbound is the Fnorm signal of the target alone; Bound is the Fnorm signal of the complex; Kd is the dissociation constant or binding affinity; and ctarget is the final concentration of target in the assay.

Excitation and MST power were optimized to ensure precise detection of real-time changes in fluorescence intensity and to record differences in fluorescence distribution during thermophoresis. Throughout the experiment, the ligand concentration was maintained at a constant level well below the Kd to ensure accurate measurements. All MST signals were normalized to account for variations in fluorescence intensity. A dose-response curve was generated with ligand concentration plotted on the horizontal axis and normalized fluorescence intensity on the vertical axis. The dissociation constant (Kd) was automatically calculated by fitting the data using the device’s built-in affinity analysis software (MO. Affinity Analysis). Data analysis and chart generation were performed using OriginPro 2024 software.

### Quantification and statistical analysis

Statistical significance was analyzed using the two-tailed Student’s t test. Differences between groups were considered to be significant when *p* < 0.05 (∗*p* < 0.05, ∗∗*p* < 0.01, ∗∗∗*p* < 0.001, ∗∗∗∗*p* < 0.0001, ns (no significance)). Error bars are indicated for duplicate or triplicate biological experiments (mean ± SD, *n* = 2 or 3), as stated in the Figure legends. All statistical analyses were conducted using Graphpad Prism 7 software.
