# Supplementary material for: Argonaute 2 inhibits RIG-I signaling via competition for viral RNA binding
Source: iScience. 2025 Aug 19;28(9):113391. doi: 10.1016/j.isci.2025.113391 (PMC12424427; doi:10.1016/j.isci.2025.113391)
Supplement: Document S1. Figures S1–S11 and Table S1 [file mmc1.pdf]

iScience, Volume *28*

Supplemental information

Argonaute 2 inhibits RIG-I signaling via competition for viral RNA binding

Honglian Liu, Yingyin Liao, Fei Yu, Leo Ngo-Shing Li, Yajie Zhang, Lin Zhu, Guangshan Xie, Jiayan Liu, Siwen Liu, Shaofeng Deng, Rachel Chun-Yee Tam, Wenjun Song, Pin Chen, Xiaofeng Huang, Conor J. Cremin, Yixin Chen, Min Zheng, Pui Wang, Zongwei Cai, Kwok-Yung Yuen, Honglin Chen, and Bobo Wing-Yee Mok

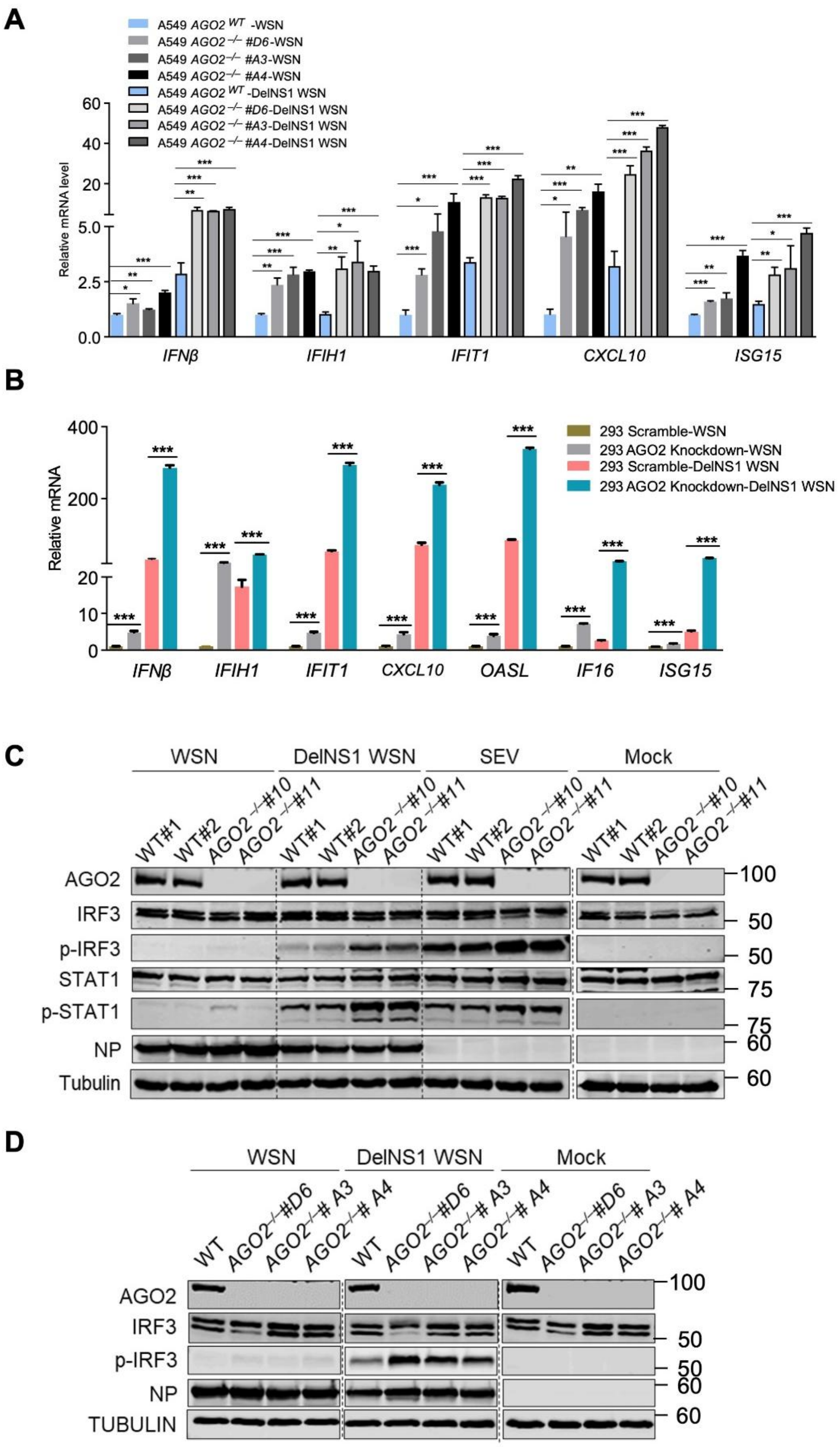
A
A549 AGO2 WT -WSN
A549 AGO2 −/− #D6-WSN
A549 AGO2 −/− #A3-WSN
A549 AGO2 −/− #A4-WSN
A549 AGO2 WT -DelNS1 WSN
A549 AGO2 −/− #D6-DelNS1 WSN
A549 AGO2 −/− #A3-DelNS1 WSN
A549 AGO2 −/− #A4-DelNS1 WSN
Relative mRNA level
IFNβ
IFIH1
IFIT1
CXCL10
ISG15
B
293 Scramble-WSN
293 AGO2 Knockdown-WSN
293 Scramble-DelNS1 WSN
293 AGO2 Knockdown-DelNS1 WSN
Relative mRNA
IFNβ
IFIH1
IFIT1
CXCL10
OASL
IF16
ISG15
C
WSN
DelNS1 WSN
SEV
Mock
WT#1
WT#2
AGO2 −/− #10
AGO2 −/− #11
AGO2
IRF3
p-IRF3
STAT1
p-STAT1
NP
Tubulin
D
WSN
DelNS1 WSN
Mock
WT
AGO2 −/− #D6
AGO2 −/− # A3
AGO2 −/− # A4
AGO2
IRF3
p-IRF3
NP
TUBULIN

Figure S1 AGO2 suppresses antiviral innate immune responses, related to Figure 1.
(A) RT-qPCR analysis of mRNA expression from antiviral genes (*IFNb*, *IFIH1*, *IFIT1*, *CXCL10* and *ISG15*) in control ($AGO2^{WT}$) and three independent $AGO2^{–/–}$ A549 clones (#D6, #A3 and #A4) that were infected with WT WSN or DelNS1 WSN at an MOI of 1 for 8 h, normalized to control ($AGO2^{WT}$) cells infected with WT WSN. (B) RT-qPCR analysis of mRNA expression from antiviral genes (*IFNb*, *IFIH1*, *IFIT1*, *CXCL10*, *OASL*, *IF16* and *ISG15*) in scramble (control) and AGO2 knockdown 293 cells that were infected with WT WSN or DelNS1 WSN at an MOI of 1 for 8 hours, normalized to scramble cells infected with WT WSN. (C) Immunoblot analysis of virus-triggered phosphorylation of IRF3 and STAT1 in two independent control $AGO2^{WT}$ (#1 and #2) and $AGO2^{–/–}$ (#10 and #11) 293 clones that were either mock-infected or infected with WT WSN or DelNS1 WSN at an MOI of 1 for 8 hours or SeV at an MOI of 1 for 4 hours. (**D**) Immunoblot analysis of virus-triggered phosphorylation of IRF3 in control ($AGO2^{WT}$) and three independent $AGO2^{–/–}$ A549 clones (#D6, #A3 and #A4) that were either mock-infected or infected with WT WSN or DelNS1 WSN at an MOI of 1 for 8 hours.

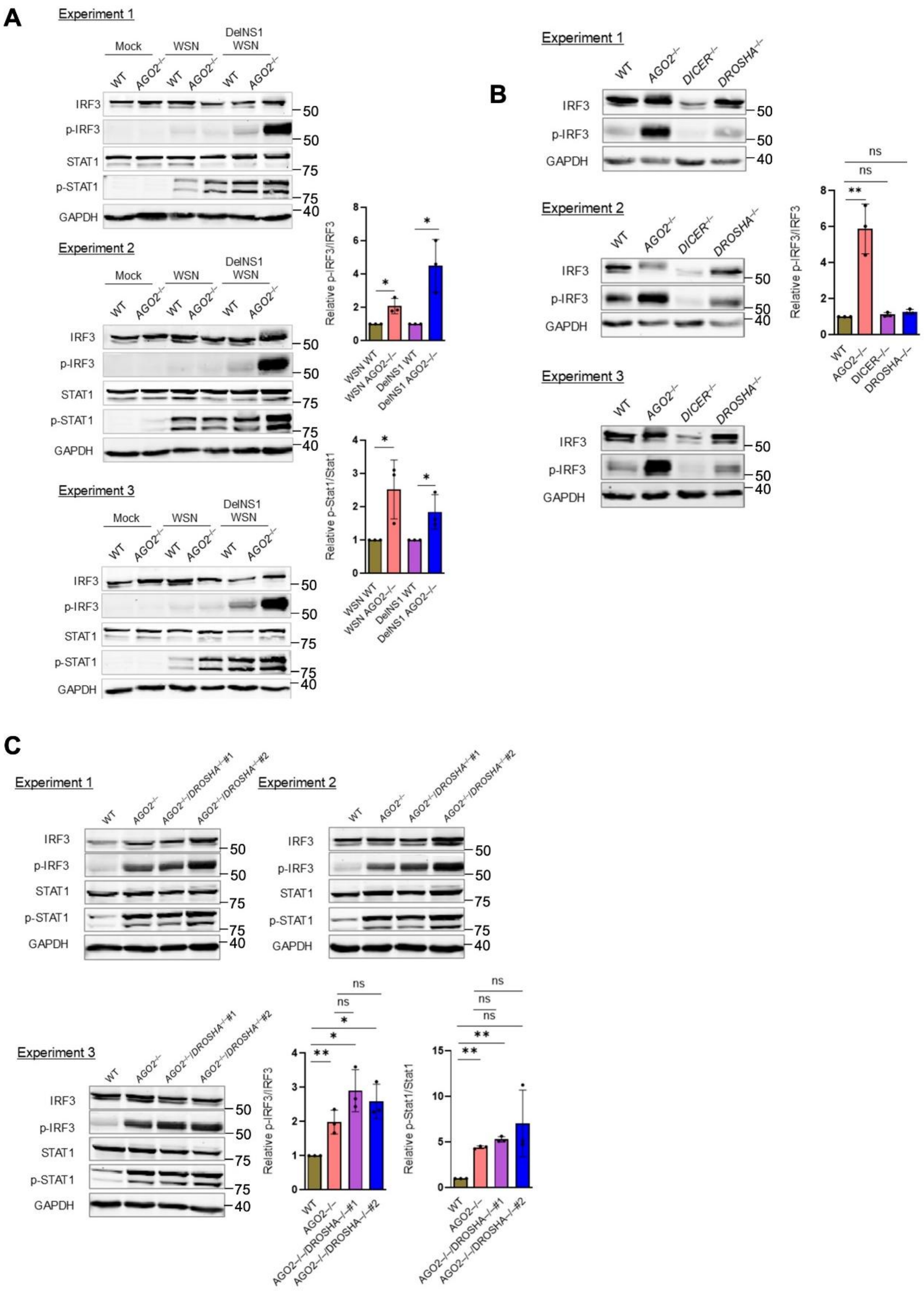

Figure S2 Quantification analysis of p-IRF3 and p-STAT1 signal for **Western Blots, related to Figure 1 and 2.**
(A-C) The experiments displayed in Figures 1B, 2A, and 2C were replicated thrice. Band quantification was conducted using AzureSpot Pro analysis software (version 1.4-583, Azure Biosystems). (Right panel) The relative expression of p-IRF3 and p-STAT1 was normalized to their respective total IRF3 and STAT1 levels. A two-tailed Student's t-test was used to analyze statistical significance. In all panels, *p < 0.05, **p < 0.01, ***p < 0.001. Error bars represent mean ± SD from biological triplicates (n=3).

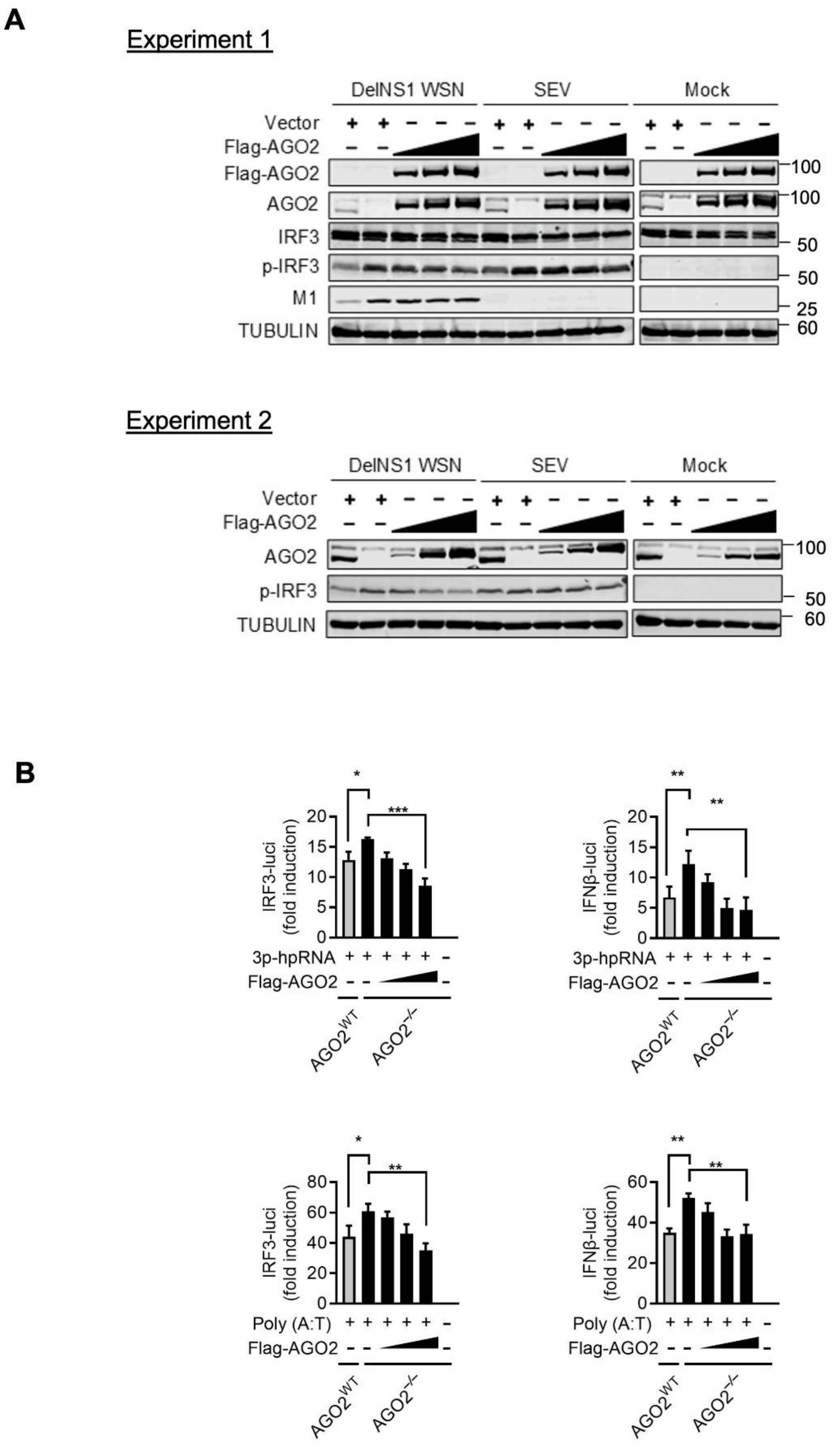

Figure S3. Reintroduction of AGO2 rescues phenotypes in KO Cells in a dose-dependent manner, related to Figure 3.
(A) The experiment shown in Figure 3A was replicated twice, confirming a consistent trend. (B) The experiment illustrated in Figure 3B was repeated using another 293 AGO2 KO clone (#11). Statistical significance was assessed using a two-tailed Student's t-test. In all panels, *p < 0.05, **p < 0.01, ***p < 0.005, and ns indicates not significant. Error bars represent the mean ± standard deviation from three biological replicates (n=3).

A

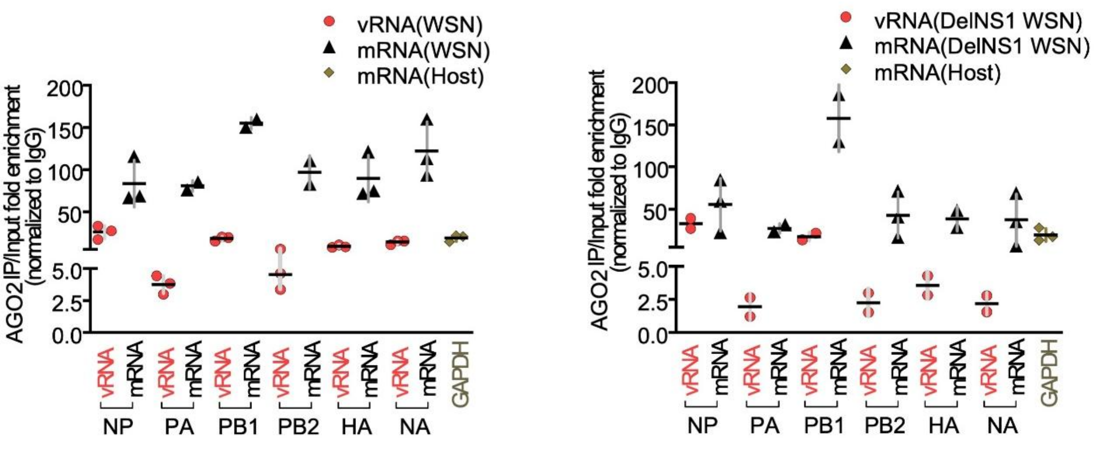

B

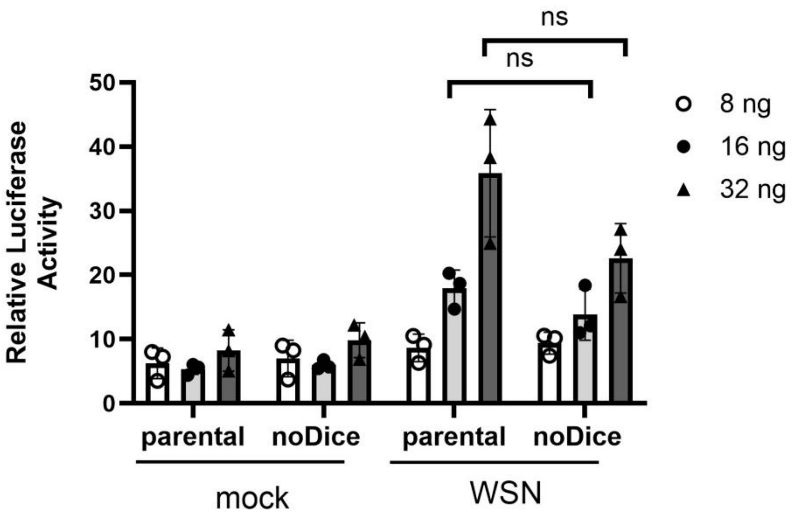

Figure S4. AGO2 associates with the viral RNA of influenza viruses independent of miRNAs, related to Figure 4.

(A) CLIP analysis of viral genomic RNA and viral mRNA conducted by endogenous Ago2 in A549 cells infected with WSN (left) or DelNS1 WSN (right) at an MOI of 1 for 8 hours (Figure 4A). (B) IFNb promoter luciferase activities with increased concentration of AGO2 precipitates obtained from mock or WSN infected 293T parental or noDice cells. Statistical significance was analyzed using a two-tailed Student's t-test. "ns" indicates no significance.

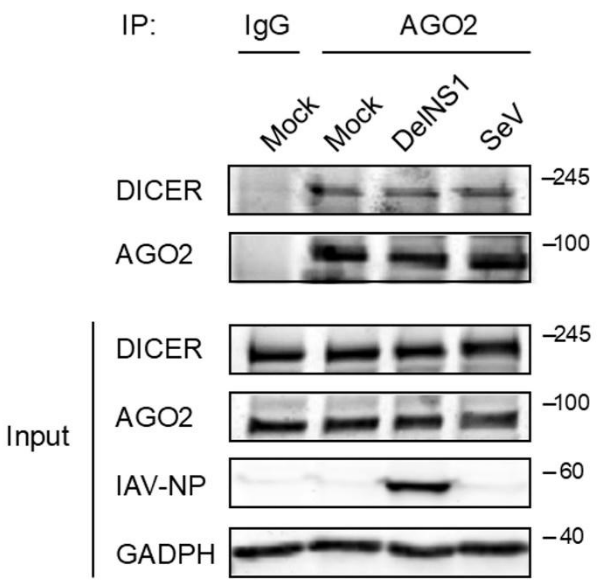

Figure S5. Interaction between AGO2 and DICER under infection, related to Figure 4.
A549 cells were either left uninfected (mock infection) or infected with DelNS1 WSN at a MOI of 1 for 8 hours, or with SEV at an MOI of 1 for 4 hours. Samples were then immunoprecipitated with an antibody against AGO2 or IgG as control for further analysis.

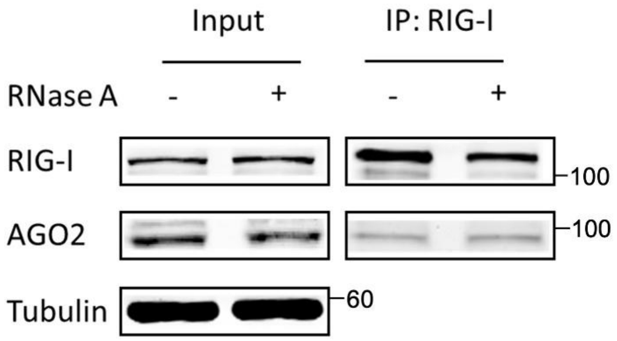

Figure S6. The effect of RNase on the binding between AGO2 and RIG-1, related to Figure 5.

A549 cells were lysed and then incubated at room temperature for 10 min without or with RNase A (100U/ml) prior to immunoprecipitation using an antibody against RIG-I.

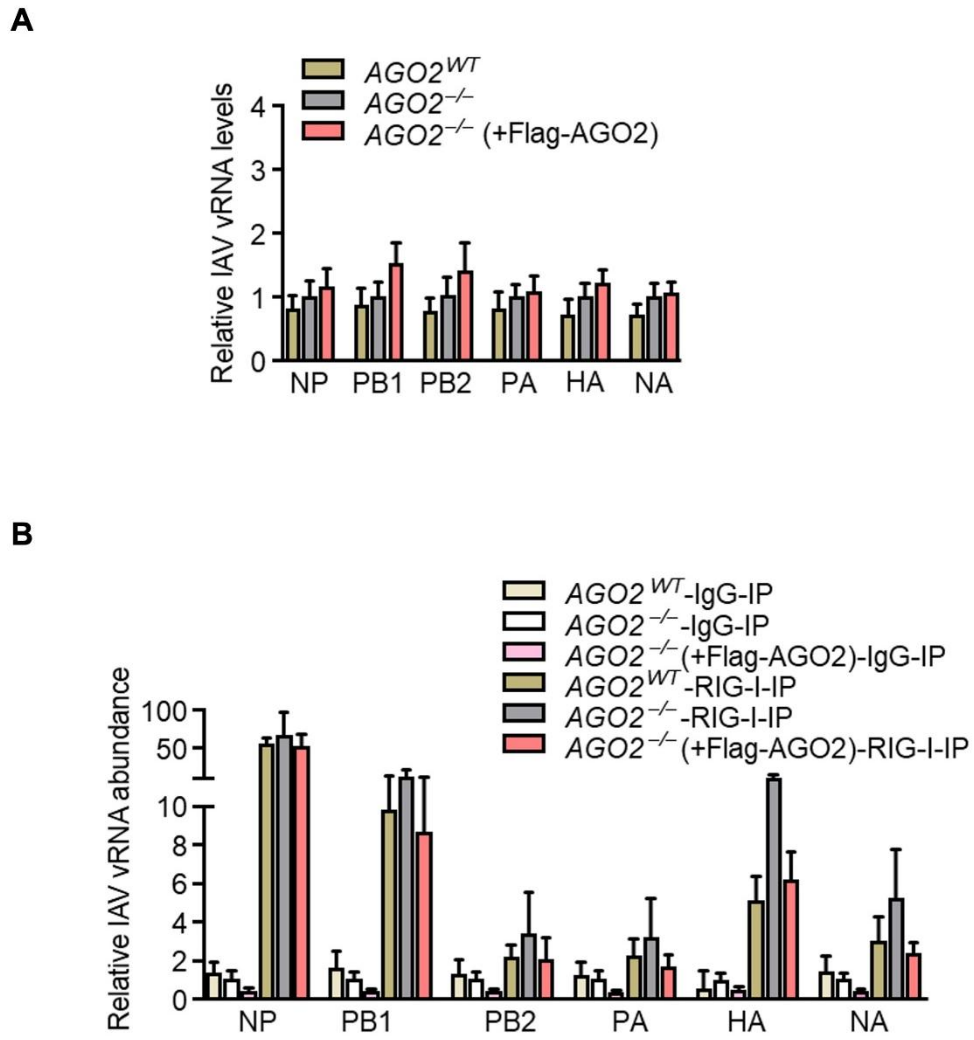

Figure S7. AGO2 interacts with RIG-I in virus-infected cells and competes to bind viral RNA containing 5'-triphosphate, related to Figure 6.

(A) RT-qPCR analysis of relative abundance of viral genomic RNA in control (*AGO2*^{WT}) 293 cells, *AGO2*$^{-/-}$293 cells and *AGO2*$^{-/-}$ 293 cells overexpressing Flag-AGO2 that had been infected with DelNS1 WSN at an MOI of 1 for 8 hours. (**B**) RT-qPCR analysis of purified RNA associated with RIG-I in control 293 cells, *AGO2*$^{-/-}$ 293 cells and *AGO2*$^{-/-}$ 293 cells overexpressing Flag-AGO2 that had been infected with DelNS1 WSN at an MOI of 1 for 8 hours; the fold change in specific target sequences was used to calculate differences in enrichment in IP samples relative to input samples and then normalized to those of control IgG. Error bars represent mean ± SD from biological triplicates (n=3).

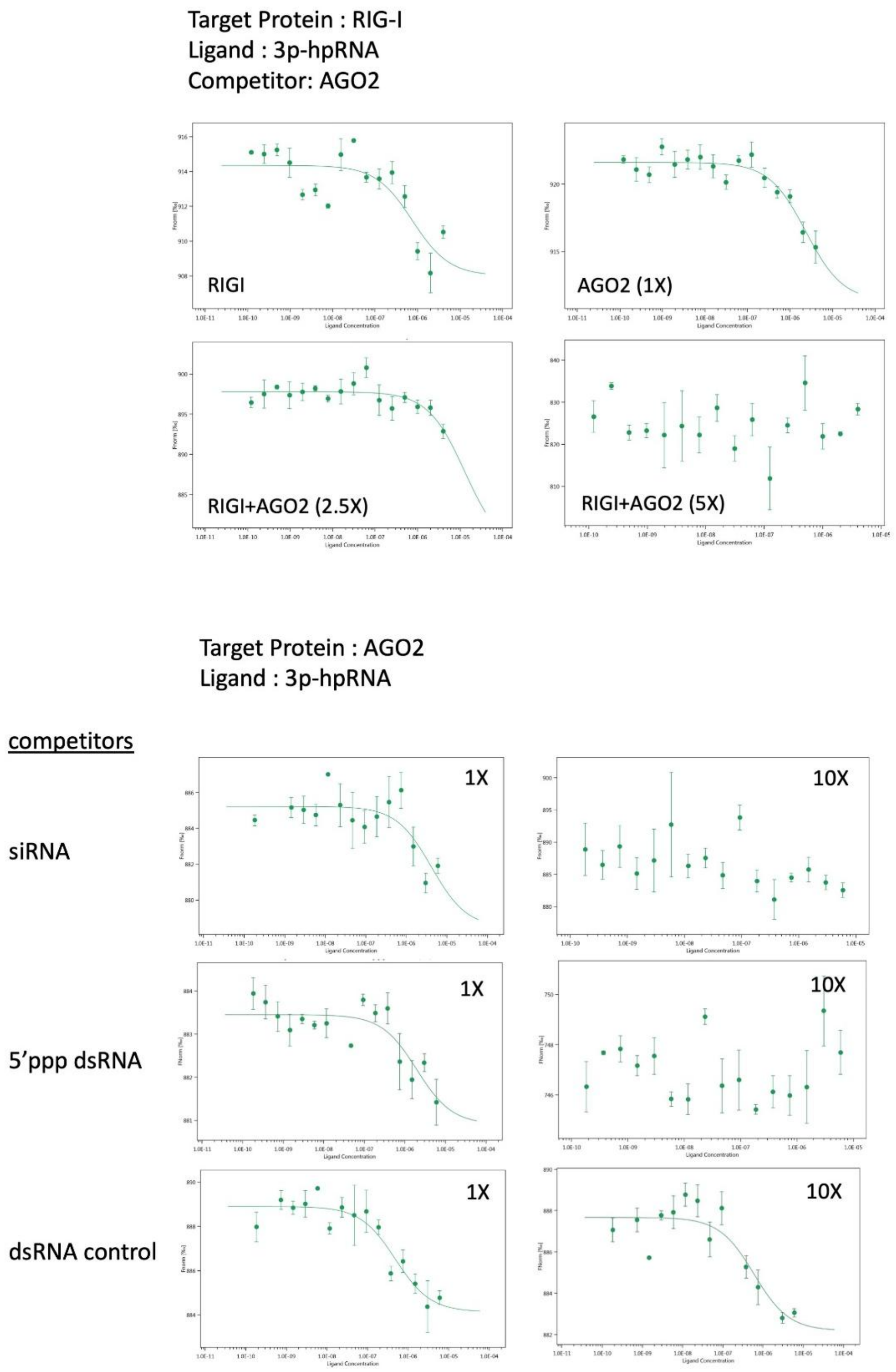

Figure S8. MST binding assay, related to Figure 7.
(A) Binding affinity of 3p-hpRNA to RIG-I was measured in the presence of increasing concentrations of AGO2 as a competitor. (B) Binding affinity of 3p-hpRNA to AGO2 was assessed using different competitors (siRNA, 5'ppp-dsRNA, and dsRNA control) at two concentrations (1X and 10X).

A

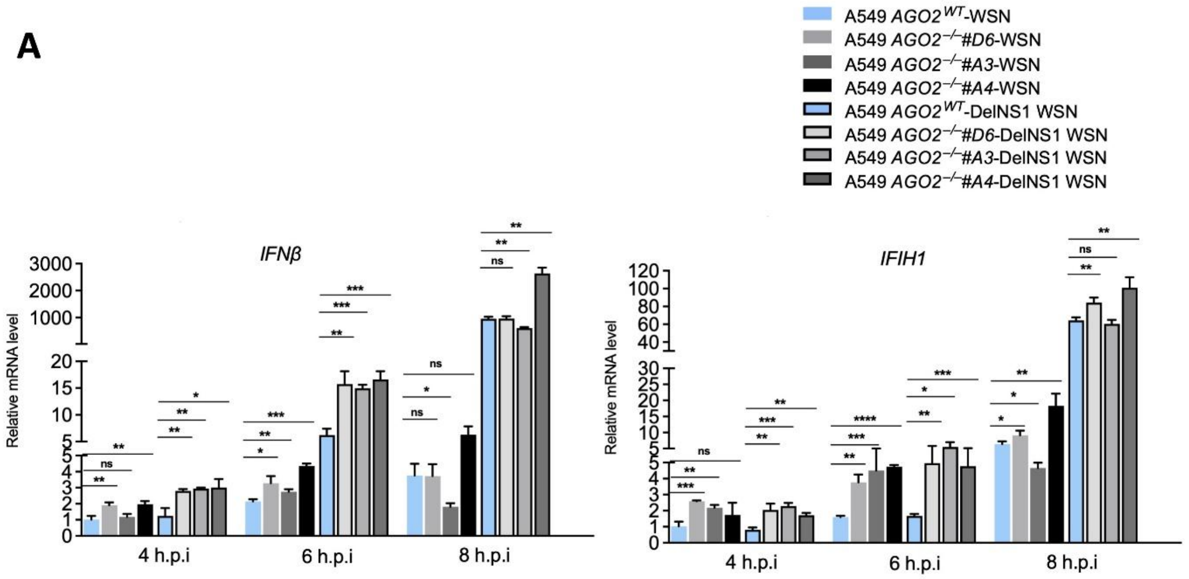
A549 AGO2^{WT}-WSN
A549 AGO2$^{-/-}$#D6-WSN
A549 AGO2$^{-/-}$#A3-WSN
A549 AGO2$^{-/-}$#A4-WSN
A549 AGO2^{WT}-DelNS1 WSN
A549 AGO2$^{-/-}$#D6-DelNS1 WSN
A549 AGO2$^{-/-}$#A3-DelNS1 WSN
A549 AGO2$^{-/-}$#A4-DelNS1 WSN
IFNβ
IFIH1
Relative mRNA level
4 h.p.i
6 h.p.i
8 h.p.i

B

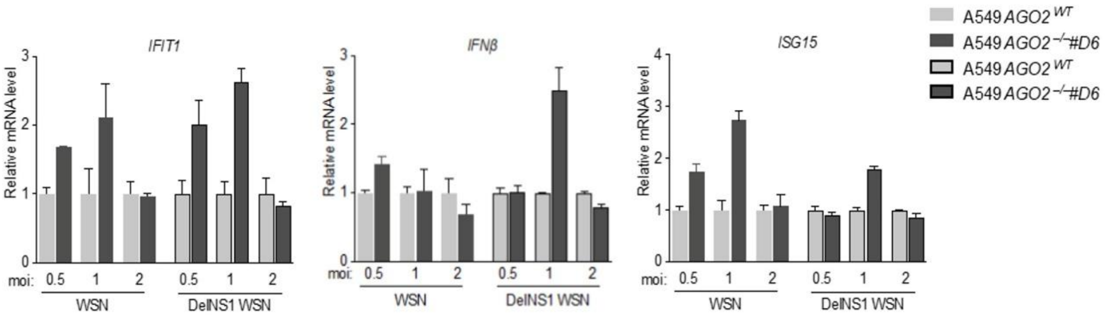
A549 AGO2^{WT}
A549 AGO2$^{-/-}$#D6
A549 AGO2^{WT}
A549 AGO2$^{-/-}$#D6
IFIT1
IFNβ
ISG15
Relative mRNA level
moi: 0.5 1 2 0.5 1 2
WSN
DelNS1 WSN

C

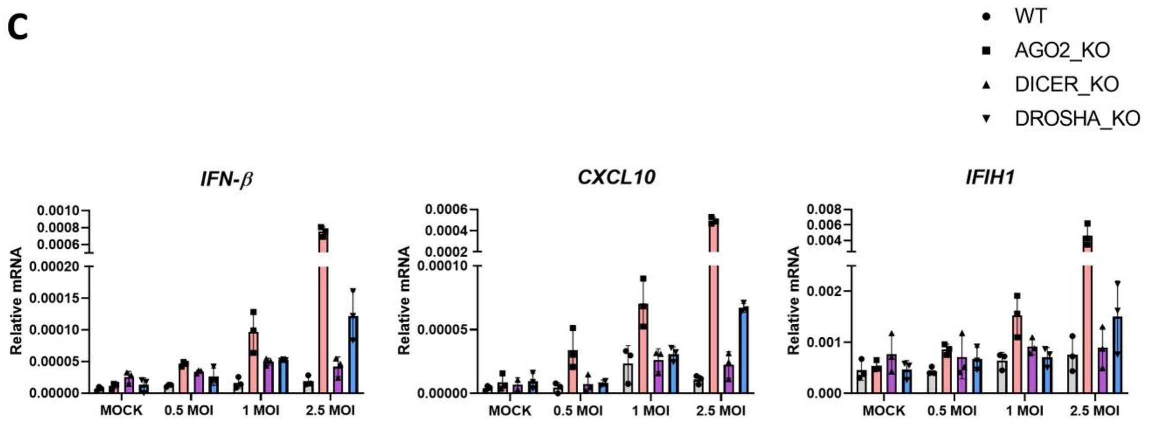
WT
AGO2_KO
DICER_KO
DROSHA_KO
IFN-β
CXCL10
IFIH1
Relative mRNA
MOCK
0.5 MOI
1 MOI
2.5 MOI

Figure S9. Optimization of infection conditions.
(A) RT-qPCR analysis of antiviral gene mRNA expression in A549 cells (*AGO2*^{WT} and *AGO2*$^{-/-}$: #D6, #A3 and #A4) infected with WSN and DelNS1 WSN at an MOI of 1 at different hours post infection (4, 6 and 8 hpi). (B) RT-qPCR analysis of antiviral gene mRNA expression in A549 cells (*AGO2*^{WT} and *AGO2*$^{-/-}$ #D6) infected with WSN and DelNS1 WSN at different MOI (0.5, 1 and 2) for 8 hours. (C) RT-qPCR analysis of mRNA expression from antiviral genes in in A549 cells (control, AGO2-KO, DICER-KO and DROSHA-KO) infected with DelNS1 WSN at different MOI (0.5, 1 and 2) for 6 hours. A two-tailed Student's t-test was used to analyze statistical significance. In all panels, *p < 0.05, **p < 0.01, ***p < 0.001. Error bars represent mean ± SD from biological triplicates (n=3).

A

Clones	Indel %	Sequence Analysis	Knockout Score
A549 AGO2 KO (A3)	-1 (90%)<br>-7 (3 %)<br>+11 (1%)	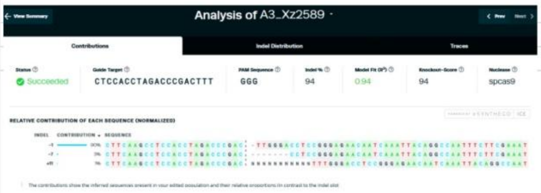	94%
A549 AGO2 KO (A4)	-2 (47%)<br>-1 (50 %)	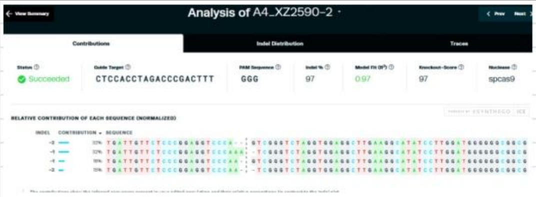	97%
A549 AGO2 KO (D6)	+1 (98%)	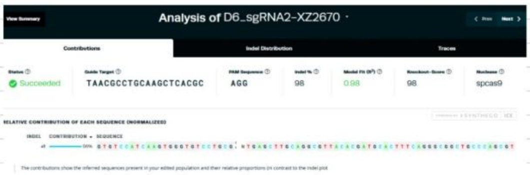	96%
293 AGO2 KO (#10)	+2 (46%)<br>-8 (44 %)	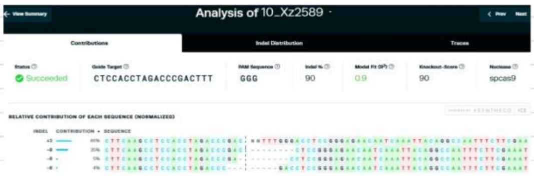	90%
293 AGO2 KO (#11)	+2 (44%)<br>-5 (42 %)	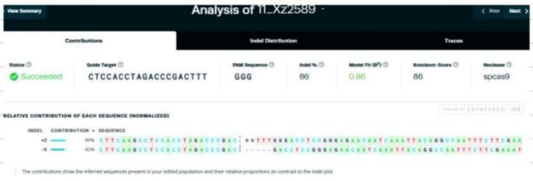	86%
A549 Dicer KO (F3)	+1 (75%)<br>-13 (9 %)<br>-13 (9%)<br>-23 (5%)<br>-13 (2%)	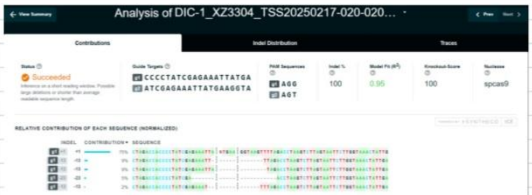	100%
A549 Drosha KO (D3)	+2 (42%)<br>+1 (38 %)<br>+18 (3%)<br>-13 (3%)<br>+15 (2%)<br>+11 (2%)<br>+9 (2%)<br>-3 (2%)<br>+13 (1%)<br>+6 (1%)<br>-30 (1%)<br>-19 (1%)<br>-4 (1%)<br>-5 (1%)	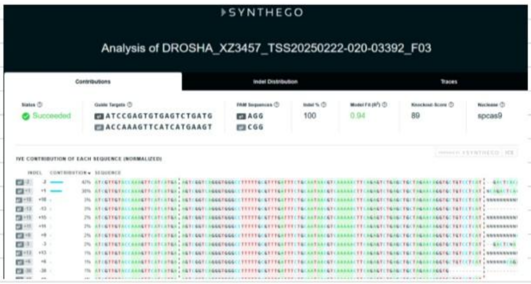	89%

B

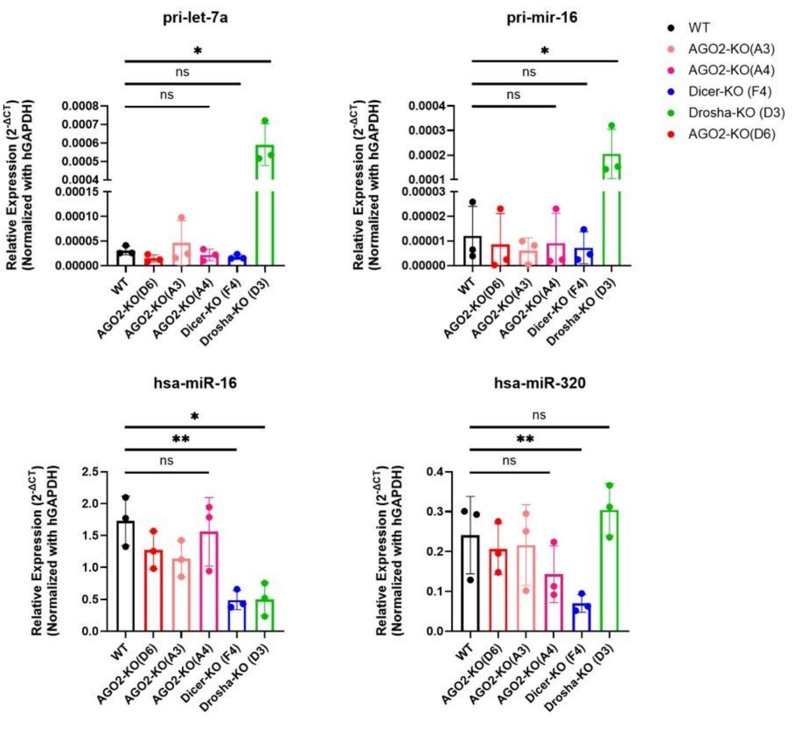

pri-let-7a
pri-mir-16
hsa-miR-16
hsa-miR-320
Relative Expression (2-ΔCT) (Normalized with hGAPDH)
ns
*
**
WT
AGO2-KO(A3)
AGO2-KO(A4)
Dicer-KO (F4)
Drosha-KO (D3)
AGO2-KO(D6)

C

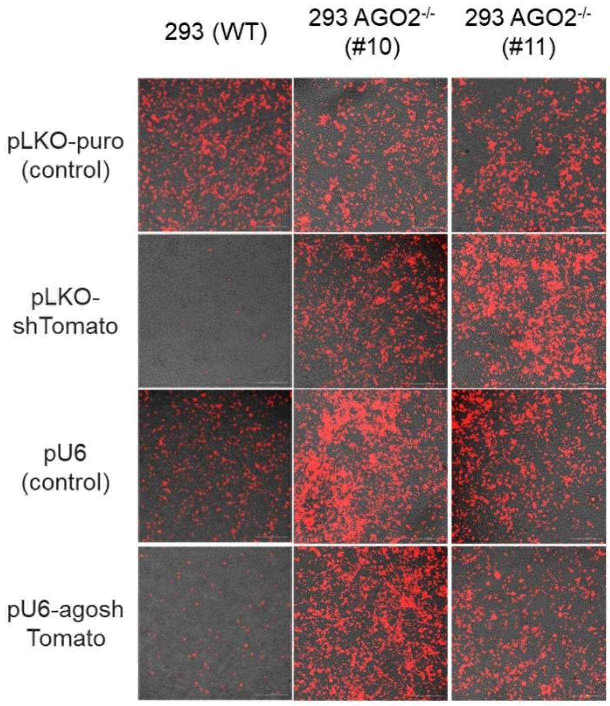

293 (WT)
293 AGO2-/- (#10)
293 AGO2-/- (#11)
pLKO-puro (control)
pLKO-shTomato
pU6 (control)
pU6-agosh Tomato

Figure S10. Genomic information and functional validation of CRISPR Knockout (KO) Clones. (A) Target genomic sequence of CRISPR KO clones employed in this study. The sequencing data was analyzed using ICE analysis (Synthego). (B) qRT-PCR analysis of pri-miRNAs (pri-mir-16 and pri-let-7a) and mature miRNAs (miR-320 and miR-16) in A549 wild-type and knockout cells. Upon DROSHA depletion, pri-mir-16a and pri-let-7a levels increase, whereas they remain constant in AGO2 and DICER KO cells (1). MiR-16 is DROSHA-dependent, while miR-320 is DROSHA-independent. In DICER KO cells, both miR-16 and miR-320 levels decrease (2). (C) Functional validation of AGO2-KO clones. 293 cells (control and AGO2 KO clones) were transfected with the tdTomato expression plasmid (BII-Ba-tdtomato), in conjunction with either (1) pU6-control or pU6-agoshRNA targeting Tomato, or (2) pLKO-control or pLKO-shRNA targeting Tomato (plasmids kindly provided by Professor SC Kwon, HKU). The knockdown of the Tomato signal was subsequently assessed and documented using Cytation 5 (3).

1. Han, J., Pedersen, J.S., Kwon, S.C., Belair, C.D., Kim, Y.K., Yeom, K.H., Yang, W.Y., Haussler, D., Blelloch, R., and Kim, V.N. (2009). Posttranscriptional crossregulation between Drosha and DGCR8. Cell *136*, 75-84. 10.1016/j.cell.2008.10.053.
2. Kim, Y.K., Kim, B., and Kim, V.N. (2016). Re-evaluation of the roles of DROSHA, Export in 5, and DICER in microRNA biogenesis. Proc Natl Acad Sci U S A *113*, E1881-1889. 10.1073/pnas.1602532113.
3. Liu, Y.P., Schopman, N.C., and Berkhout, B. (2013). Dicer-independent processing of short hairpin RNAs. Nucleic Acids Res *41*, 3723-3733. 10.1093/nar/gkt036.

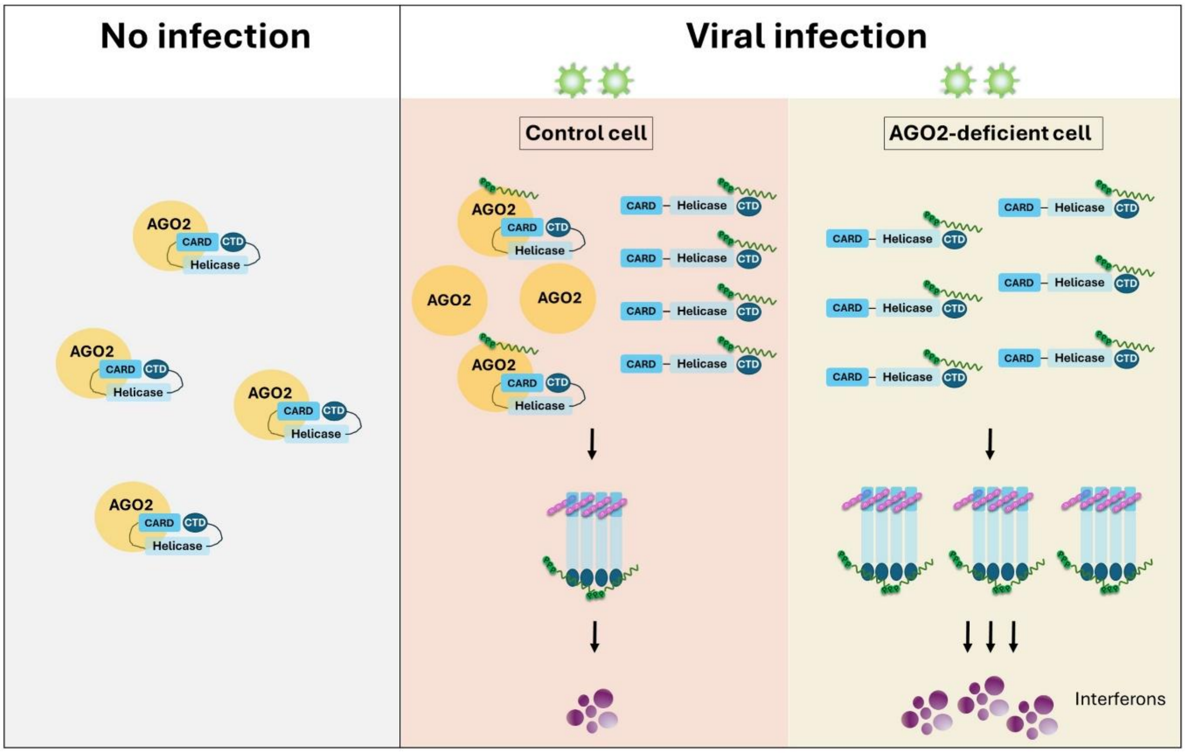

Figure S11. **Model figure**

Table S1. Primers and probes used in this study

Name	sequence
Uni-12	5′-ACGCGTGATCAGCAAAAGCAGG
gRNA sequence	Ago2: gRNA1--TAACGCCTGCAAGCTCACGC<br>gRNA2 -CTCCACCTAGACCCGACTTT<br>Dicer: gRNA1-TACCTTCATAATTTCTCGAT<br>gRNA2- CCCCTATCGAGAAATTATGA<br>Drosha: gRNA1-ACCAAAGTTCATCATGAAGT<br>gRNA2-ATCCGAGTGTGAGTCTGATG
Sequencing primers for target regions (sgRNA sites) gRNA site	Ago2_gRNA1: F- GCTAGTGGTTGGCTAGAGGC<br>R- CACAGAAAACCCACGTGACC<br>Ago2_gRNA2 - F- ACGCCTCCTCTGTGGAATGC<br>R- CGCAGACCACTTACACAGGTC<br>Dicer: gRNA1+2 – F- CATTATGACTTGCTATGTCGCC<br>R- GCAGCCAAACTCCCAATATTG<br>Drosha: gRNA1+2 – F- CCAGTTTGTTAATTCCATCAATTTC<br>R- CCATTACTCCTCTCCCCAACA
Ago2 (RT-qPCR)	F- CCAGTCACCAAACATTCCCG<br>R- CAGGGGAAGGTAGGTGTGTT
IFNβ (RT-qPCR)	F- TCTGGCACAACAGGTAGTAGGC<br>R- GAGAAGCACAACAGGAGAGCAA
CXCL10 (RT-qPCR)	F- GTGGCATTCAAGGAGTACCTC<br>R- TGATGGCCTTCGATTCTGGATT
IFIT1 (RT-qPCR)	F- TTCGGAGAAAGGCATTAGA<br>R- TCCAGGGCTTCATTCATAT
IFIH1 (RT-qPCR)	F- TCACAAGTTGATGGTCCTCAAGT<br>R- CTGATGAGTTATTCTCCATGCCC
OASL (RT-qPCR)	F- CTGATGCAGGAACTGTATAGCAC<br>R- CACAGCGTCTAGCACCTCTT
IF16 (RT-qPCR)	F- GGTCTGCGATCCTGAATGGG<br>R- TCACTATCGAGATACTTGTGGGT
ISG15 (RT-qPCR)	F- CGCAGATCACCCAGAAGATCG<br>R- TTCGTCGCATTTGTCCACCA
IL-6 (RT-qPCR)	F- AACCTGAACCTTCCAAAGATGG<br>R- TCTGGCTTGTTCCTCACTACT
TNFα (RT-qPCR)	F- ATGAGCACTGAAAGCATGATCC<br>R- GAGGGCTGATTAGAGAGAGGTC
GAPDH (RT-qPCR)	F- GCAAATTCCATGGCACCGT<br>R- GCCCCACTTGATTTTGGAGG
HMGA2 (RT-qPCR)	F- TCCCTCTAAAGCAGCTCAAAA<br>R- ACTTGTTGTGGCCATTTCCT
WSN-NP (RT-qPCR)	F- GGTGAGAATGGACGGAGAAC<br>R- CCGGCTCTCTCTCACTTGAT
WSN-PB1 (RT-qPCR)	F- CAAACACCGAAACTGGAGCA<br>R- TTGTCCACTCGTGTTTGCTG
WSN-PB2 (RT-qPCR)	F- GGAACATGCTGGGAACAGAT<br>R- TTGGGTTCTGCCTAAGGATG
WSN-PA (RT-qPCR)	F- TCTCAGCGGTCCAAATTCCT<br>R- TCTGCCAGTACTTGCTTCCA
WSN-NA (RT-qPCR)	F- CCTGATACCGGCAAAGTGAT<br>R- TTTGCTCCATCAGCAGACAC
WSN-HA (RT-qPCR)	F- ACCCGTCTAGCAGTGATGAG<br>R- GGGTTCTAGCAAGGTCCAGT
pri-let-7a (RT-qPCR)	F-TGGGATGAGGTAGTAGGTTGTA<br>R-CTATCACGTTAGGAAAGACAGTAGAT
pri-mir-16 (RT-PCR)	F-CCTTACTTCAGCAGCACAGTTA<br>R-TGCCTTAGCAGCACGTAAATA

hGAPDH (RT-PCR)	F-ACCCAGAAGACTGTGGATGG<br>R-CAGTGAGCTTCCCGTTCAG
